# A Small‐Molecule DEPTAC Rescues Cognitive Deficits by Targeted Dephosphorylation of Pathological Tau

**DOI:** 10.1002/advs.77088

**Published:** 2026-08-11

**Authors:** Fei Sun, Yang Liu, Qiuzhi Zhou, Qingyuan Hu, Weixia Wang, Yue Xiao, Jingfen Su, Guanting Liu, Miaomiao Sun, Lixia Chen, Hua Li, Ying Yang, Jian‐Zhi Wang

**Affiliations:** ^1^ Department of Pathophysiology School of Basic Medicine Key Laboratory of Education Ministry of China/Hubei Province for Neurological Disorders Tongji Medical College Huazhong University of Science and Technology Wuhan China; ^2^ Institute For Translational Neuroscience Nantong First People's Hospital Southeast University Nantong Jiangsu China; ^3^ Institute of Structural Pharmacology & TCM Chemical Biology Fujian Key Laboratory of Chinese Materia Medica College of Pharmacy Fujian University of Traditional Chinese Medicine Fuzhou China; ^4^ Wuya College of Innovation School of Pharmacy Key Laboratory of Structure‐Based Drug Design & Discovery Ministry of Education Shenyang Pharmaceutical University Shenyang China; ^5^ Department of Rehabilitation Tongji Hospital Tongji Medical College Huazhong University of Science and Technology Wuhan China; ^6^ Department of Clinical Medicine School of Medicine Nanchang University Nanchang China; ^7^ Department of Pathology The First Affiliated Hospital of Zhengzhou University Zhengzhou China

**Keywords:** Alzheimer's disease, DEPTAC, PP2A, targeted dephosphorylation, Tau, TP2

## Abstract

Accumulation of hyperphosphorylated Tau (p‐Tau) is a central driver of neurodegeneration in Alzheimer's disease (AD). Addressing the limitations of current therapeutics, we developed TP2, a small‐molecule DEPhosphorylation‐TArgeting Chimera (DEPTAC). TP2 is engineered to physically tether endogenous PP2A to Tau, facilitating targeted dephosphorylation. Mechanistically, TP2 induces ternary complex formation and potently reduces p‐Tau in neurons. Systemic administration of TP2 in distinct tauopathy mouse models (P301L and AAV‐hTau‐N368) significantly lowered brain p‐Tau levels, oligomers, and neurofibrillary tangles without altering global PP2A activity. Crucially, by rebalancing the phosphorylation of Tau, TP2 restored Tau's physiological function, as evidenced by the preservation of neuronal morphology, synaptic integrity, and microtubule stability. TP2 also ameliorated the dysregulated neuroimmune microenvironment. Consequently, TP2 treatment robustly reversed cognitive deficits in multiple behavioral paradigms. These findings establish TP2 as a promising therapeutic candidate that targets upstream pathological phosphorylation to halt neurodegeneration and restore cognitive function.

## Graphical abstract

TP2 is a fully synthetic small‐molecule dephosphorylation‐targeting chimera that recruits endogenous PP2A‐Bα to Tau, enabling targeted removal of pathological phosphate modifications. In two tauopathy mouse models, systemic TP2 reduces Tau pathology, preserves neuronal and synaptic integrity, improves neuroimmune homeostasis, and rescues cognition, highlighting proximity‐induced dephosphorylation as a potential therapeutic strategy for tauopathies.

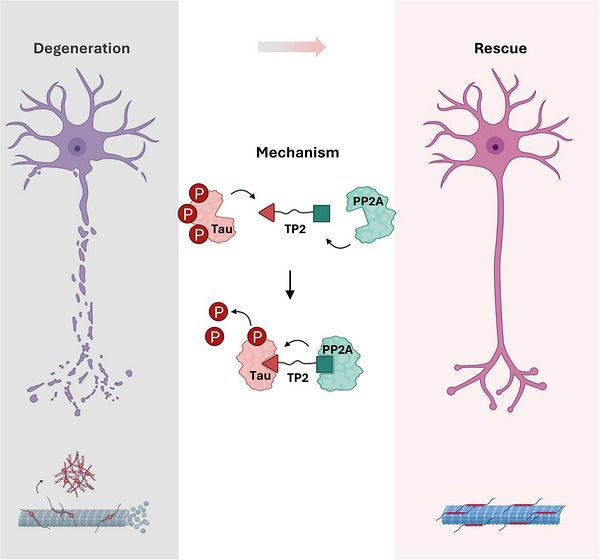

## 1 Introduction

The intraneuronal accumulation of neurofibrillary tangles (NFTs), composed primarily of hyperphosphorylated Tau (p‐Tau), is a central pathological hallmark of Alzheimer's disease (AD) and a defining feature of a class of neurodegenerative disorders known as tauopathies [1]. The hyperphosphorylation of Tau is a critical initiating event that disrupts its normal function in microtubule stabilization, promotes its misfolding and aggregation into NFTs, and ultimately drives a neurotoxic cascade leading to synaptic dysfunction and neuronal loss [2]. Consequently, targeting the pathological phosphorylation of Tau represents a key therapeutic strategy for AD and related tauopathies.

Several therapeutic avenues targeting Tau have been extensively explored, yet, as highlighted in recent comprehensive reviews, these first‐generation approaches have largely failed to translate into clinical benefits [3]. For instance, immunotherapies designed to clear extracellular Tau seeds have consistently failed to meet primary endpoints [4, 5, 6, 7]. These failures have raised fundamental questions about the therapeutic hypothesis itself, including whether targeting extracellular Tau is the correct strategy given its predominantly intracellular pathology, and whether sufficient antibody concentrations can even be achieved across the blood‐brain barrier. Similarly, small‐molecule inhibitors of Tau aggregation have been plagued by poor brain bioavailability and the lack of demonstrated target engagement in vivo [8, 9]. Direct modulation of Tau's post‐translational state by targeting key kinases, such as GSK‐3β or PP2A, has also proven unsuccessful [10, 11]. The primary obstacle lies in the ubiquitous and pleiotropic nature of these enzymes; their inhibition inevitably disrupts numerous critical cellular pathways, leading to prohibitive off‐target toxicity.

While approaches aiming to reduce total Tau expression with antisense oligonucleotides (ASOs) are arguably the most promising to date, showing encouraging reduction of cerebrospinal fluid Tau in patients [12]—they are not without significant drawbacks. A major conceptual disadvantage is their indiscriminate reduction of all Tau isoforms, which risks compromising the vital physiological functions of healthy Tau in microtubule stabilization and axonal transport [13, 14]. Furthermore, their reliance on invasive intrathecal delivery poses a considerable burden for long‐term treatment [12]. Collectively, the specific shortcomings of these diverse strategies converge on a single point: the urgent need for a therapeutic paradigm that can selectively neutralize pathogenic, hyperphosphorylated Tau while sparing its physiological counterpart and avoiding systemic toxicity.

To address this challenge, our group previously pioneered the concept of DEPhosphorylation TArgeting Chimeras (DEPTACs) [15]. Unlike proteolysis‐targeting chimeras (PROTACs), which typically drive the degradation of the target protein regardless of its phosphorylation state, thereby risking the depletion of physiological Tau essential for microtubule stability [16, 17], DEPTACs achieve pathology‐specific correction by recruiting endogenous phosphatases to selectively dephosphorylate hyperphosphorylated Tau, thereby restoring its native functional state and facilitating its physiological clearance without forcing non‐selective proteolysis. Our initial peptide‐based DEPTACs have demonstrated remarkable efficacy in clearing p‐Tau and rescuing cognitive deficits in multiple preclinical tauopathy models [15, 18, 19, 20].

Building on this foundation, we herein report TP2, a first‐in‐class, fully synthetic small‐molecule DEPTAC specifically designed to recruit PP2A‐Bα to Tau for targeted dephosphorylation. Among the protein phosphatases that dephosphorylate Tau, PP2A is the predominant Tau‐directed phosphatase, accounting for approximately 71% of the total Tau phosphatase activity in human brain, substantially exceeding the contributions of PP1, PP5, and PP2B [21]. In particular, the Bα‐containing PP2A holoenzyme directly binds Tau through a region overlapping its microtubule‐binding domain and acts as the major cellular Tau phosphatase [22, 23], and dysregulation of the PP2A‐Bα subunit is closely linked to pathological Tau hyperphosphorylation in Alzheimer's disease [24]. Moreover, recruitment of PP2A‐Bα has been validated in our previous peptide‐based DEPTAC studies as an effective strategy for promoting Tau dephosphorylation and ameliorating Tau‐related pathology [15, 18]. We therefore selected PP2A‐Bα as the phosphatase‐recruiting effector for the development of TP2.

In this study, TP2 robustly reduces p‐Tau levels in vitro and in vivo, and significantly ameliorates cognitive impairments in both the P301L model of familial frontotemporal dementia and the AAV‐hTau‐N368 model, which recapitulates fragment‐induced pathology characteristic of sporadic AD.

## 2 Materials and Methods

### 2.1 Compounds and Reagents

TP2 was synthesized and purified by the groups of Hua Li and Lixia Chen at Fujian University of Traditional Chinese Medicine and Shenyang Pharmaceutical University, respectively. The purity of TP2 was confirmed to be >98% by HPLC analysis. Detailed synthetic procedures and characterization data are provided in the Supporting Information. TP2 was dissolved in DMSO to prepare 20 mm stock solution. Recombinant human full‐length GST‐Tau (2N4R) and His‐PP2A‐Bα subunit proteins were purchased from QYAOBIO. The K18 Tau fragment was purified in‐house by our lab.

### 2.2 Animals

Male P301L mutant transgenic mice (rTg4510) and non‐transgenic littermates were bred in‐house by our laboratory. Treatment of P301L mice was initiated at 12 months of age. Male C57BL/6J mice (8 weeks old) were purchased from Wuhan MouBaiLi Biotechnology Co., Ltd. Mice were housed under standard conditions with a 12‐h light/dark cycle and ad libitum access to food and water.

For the AAV‐hTau‐N368 model, 8‐week‐old male C57BL/6J mice received bilateral stereotaxic injections of AAV‐SYN‐EGFP‐2A‐hTau‐N368 into the hippocampal dentate gyrus (DG) at the following coordinates: anteroposterior, −1.94 mm and mediolateral, ±1.3 mm from bregma; dorsoventral, −1.75 mm from the skull. Injections were performed using an automatic microinjection system (World Precision Instruments, USA). After 4 weeks of viral expression, systemic TP2 or vehicle treatment was initiated when the mice were 3 months old.

All animal‐related experiments were approved by the Animal Care and Use Committee of Huazhong University of Science and Technology.

### 2.3 Drug Administration and Behavioral Tests

Mice (P301L, AAV‐hTau‐N368, and controls) received systemic treatment with TP2 (2.5 mg/kg) or saline vehicle via intravenous (i.v.) tail vein injection. The regimen consisted of one injection every 4 days for seven total doses. Behavioral tests were initiated 2 days after the final administration.

The Novel Object Recognition (NOR) task consisted of habituation, training, and testing phases. Mice were habituated to the empty arena for 5 min (24 h pre‐training). During training, animals explored two identical objects for 10 min. In the testing phase (24 h later), one familiar object was replaced with a novel one. Exploration time was recorded for 10 min. The discrimination index (DI) was calculated as: (Time Novel – Time Familiar) / Total Exploration Time.

Spatial memory was assessed by Morris Water Maze (MWM). In acquisition period, mice were trained for six consecutive days (3 trials/day). In each trial, mice were allowed 60 s to locate the hidden platform. Those failing to find it were guided to the platform and allowed to stay for 30 s. Escape latency was recorded. In probe trial, 1 day after the last training, the platform was removed. Mice swam freely for 60 s. Latency to reach the target zone and platform site crossings were quantified to assess memory retention.

For Contextual Fear Conditioning (CFC), mice were placed in a conditioning chamber. Following a 3‐min habituation, three foot‐shocks (0.9 mA, 3 s) were delivered at 1‐min intervals. 24 h later, mice were re‐exposed to the same chamber for 3 min without shock. Freezing behavior (complete immobility) was recorded and quantified as an index of associative memory.

### 2.4 Primary Neuron Culture and Cell Lines

Primary hippocampal neurons were isolated from embryonic day 18 (E18) Sprague‐Dawley rat embryos. Hippocampi were dissected, dissociated with trypsin, and plated onto poly‐D‐lysine‐coated plates in Neurobasal medium supplemented with B27, GlutaMAX, and penicillin/streptomycin (Gibco). Half of the medium was replaced every 3 days. Experiments were performed at days in vitro (DIV) 10–12. HEK293 and HEK293‐Tau cells were cultured in DMEM supplemented with 10% fetal bovine serum (FBS) and antibiotics at 37°C in a humidified 5% CO_2_ atmosphere.

### 2.5 Surface Plasmon Resonance (SPR)

Direct binding of TP2 to GST‐Tau and His‐PP2A‐Bα, as well as TP2‐induced ternary complex formation, was measured by SPR using a Biacore 8K instrument (Cytiva). GST‐Tau and His‐PP2A‐Bα were each diluted to 50 µg/mL in sodium acetate buffer and immobilized via amine coupling onto separate flow cells of a CM5 sensor chip (Cytiva) at 10 µL/min, following activation with EDC/NHS and blocking with ethanolamine; reference flow cells underwent the same activation and blocking steps without protein immobilization. Final immobilization levels were 11,785 RU for GST‐Tau and 6,650 RU for His‐PP2A‐Bα.

TP2 was serially diluted in running buffer (1× PBS‐P+, pH 7.4, 5% (v/v) DMSO) and injected over the immobilized surfaces at 30 µL/min, from low to high concentration (0.3125–10 µm over GST‐Tau; 3.125–100 µm over His‐PP2A‐Bα). For the ternary complex assay, TP2 (0.3125–10 µm) combined with 1 µm His‐PP2A‐Bα was injected over immobilized GST‐Tau. The chip was regenerated with 10 mm glycine‐HCl (pH 2.0) for 5 min between injections. A solvent correction curve (4.5–5.8% DMSO series) was applied to account for bulk DMSO refractive index effects. Sensorgrams were globally fitted to a 1:1 Langmuir binding model using Biacore Insight evaluation software (Cytiva) to obtain association (k_a_) and dissociation (k_d_) rate constants and the equilibrium dissociation constant (K_D_).

### 2.6 Fluorescence Lifetime Imaging Microscopy (FLIM‐FRET)

HEK293 cells were co‐transfected with plasmids encoding GFP‐Tau (FRET donor) and mCherry‐PP2A‐Bα (FRET acceptor) using Lipofectamine 3000. Twenty‐four hours post‐transfection, cells were treated with 5 µm TP2 or vehicle for 6 h. FLIM imaging was performed using a Stellaris STED (Leica) equipped with a pulsed laser. The fluorescence lifetime of the GFP donor was measured, and FRET efficiency was calculated based on the reduction in donor lifetime in the presence of the acceptor compared to the donor‐only control.

### 2.7 Preparation of K18 Fibrils

Recombinant K18 protein (50 µm) was fibrillized by incubation with heparin (12.5 µm) in a buffer containing 10 mm HEPES, 100 mm NaCl, and 2.5 mm DTT (pH 7.4) at 37°C for 7 days, with fresh DTT supplemented daily. Following incubation, the mixture was ultracentrifuged at 112 817 × g for 30 min at 25°C. The resulting pellet was resuspended in 10 mm phosphate buffer (pH 6.0) and sonicated for five cycles (15 s on, 10 s off) to generate seed‐competent fibrils.

### 2.8 Cell Viability Assay

Primary neurons were treated with TP2 (0–20 µm) for 24 h. Cell viability was assessed using CCK‐8 kit (Biosharp, BS350B) by measuring absorbance at 450 nm.

### 2.9 Co‐Immunoprecipitation (Co‐IP) and Western Blotting

Primary neurons were lysed in IP lysis buffer (Beyotime, P0013) containing protease and phosphatase inhibitors. Lysates were incubated with anti‐Tau (Santa Cruz, #sc‐58860, 1:100) antibody overnight at 4°C, followed by incubation with Protein A/G magnetic beads. Immune complexes were washed, eluted with SDS sample buffer, and analyzed by Western blotting.

For Western Blotting, brain tissues or cells were homogenized in RIPA buffer. Protein concentrations were determined using a BCA assay. Equal amounts of protein were separated by SDS‐PAGE and transferred to NC membranes. Membranes were blocked and incubated with primary antibodies against anti‐PP2A B Subunit (Cell Signaling Technology, #4953, 1:1000), anti‐pT181 (Signalway Antibody, #11107, 1:1000), anti‐pS199 (Invitrogen, #44734G, 1:1000), anti‐pS262 (Invitrogen, #44‐750G, 1:1000), anti‐pS396 (Signalway Antibody, #11102, 1:1000), anti‐pS404 (Signalway Antibody, #11112, 1:1000), AT8 (pS202/T205, Thermo Fisher Scientific, #MN1020, 1:1000), anti‐Tau‐N368 (developed by AtaGenix, Wuhan, 1:1000), Tau5 (Santa Cruz, #sc‐58860, 1:1000), HT7 (Invitrogen, #MN1000, 1:1000), anti‐Synapsin I (SYN, ABclonal, #A24122, 1:1000), anti‐Synaptophysin (SYP, ABclonal, #A6344, 1:1000), anti‐PSD95 (ABclonal, #A0131, 1:1000), anti‐GluA2 (ABclonal, #A11316, 1:1000), anti‐GluN2A (ABclonal, #A0924, 1:1000), anti‐GluN2B (ABclonal, #A27739, 1:1000), anti‐β‐actin (Abclonal, #AC026, 1:5000). Secondary antibodies utilized were: HRP‐conjugated Goat Anti‐Rabbit IgG (Beyotime, #A0208, 1:3000), HRP‐conjugated Goat Anti‐Mouse IgG (Beyotime, #A0216, 1:3000), HRP‐conjugated Mouse anti‐Rabbit IgG Light Chain mAb (Abclonal, AS126). Bands were visualized using ECL and quantified with ImageJ software.

### 2.10 Insoluble Tau Extraction

Tissue samples were homogenized in inhibitor‐containing RIPA buffer (50 mm Tris‐HCl, 100 mm NaCl, 1% Triton X‐100, 5 mm EDTA, 1:100 PMSF) and centrifuged at 12 000 × g (20 min, 4°C) to separate the RIPA‐soluble supernatant. The resulting pellets were washed and dissolved in 2% SDS buffer via incubation at 37°C and sonication (60 pulses), producing the RIPA‐insoluble fraction.

### 2.11 PP2A Phosphatase Activity Assay

PP2A activity was assessed using a PP2A Immunoprecipitation Phosphatase Assay Kit (Millipore, #17‐313). Hippocampi were lysed in phosphate‐free buffer (20 mm imidazole‐HCl, pH 7.0, 2 mm EGTA, 2 mm EDTA, protease inhibitors) and centrifuged (2000 × g, 5 min) to remove debris. The supernatants were immunoprecipitated with 4 µg of anti‐PP2A C subunit antibody (clone 1D6) and Protein A agarose beads for 2 h at 4°C. Beads were washed and incubated with 750 µm threonine phosphopeptide substrate (K‐R‐pT‐I‐R‐R) at 30°C for 10 min. Phosphate release was detected using Malachite Green Solution and measured at 650 nm. Activity was normalized to total protein concentration.

### 2.12 Immunostaining and Histology

Following anesthesia, mice were perfused with saline and 4% paraformaldehyde. Brains were post‐fixed overnight, cryoprotected in graded sucrose (20%–30%), and sectioned coronally (30 µm) using a cryostat (Leica).

For Immunofluorescence, free‐floating sections were blocked (5% BSA, 0.3% Triton X‐100) and incubated overnight at 4°C with primary antibodies. Sections were subsequently labeled with Alexa Fluor‐conjugated secondary antibodies (Invitrogen, 1:500) and counterstained with DAPI. For Immunohistochemistry, all the procedures followed the instructions of IHC Detect Kit (Proteintech, #PK10006). The following primary antibodies were used: MAP2 (Proteintech, #17490‐1‐AP, 1:500), T22 (Millipore, #ABN454, 1:500), NeuN (CST, #94403, 1:200), IBA1 (Wako, #019‐19741, 1:500), GFAP (CST, #12389, 1:400).

Neurofibrillary tangles were detected using a modified Gallyas silver protocol as previously described [25]. Briefly, sections mounted on gelatin‐coated slides were treated sequentially with 5% periodic acid (5 min) and alkaline silver iodide (1 min). Following a 0.5% acetic acid wash, sections were developed, fixed, dehydrated, cleared in xylene, and cover slipped.

Dendritic architecture was visualized using the FD Rapid GolgiStain Kit (FD NeuroTechnologies). Fresh brains were impregnated for 2 weeks, sectioned at 100 µm using a vibratome, and developed. Pyramidal neurons in the CA1 region were imaged to quantify dendritic complexity (Sholl analysis) and spine density (spines/10 µm).

Images were acquired via an automated slide scanner (VS200, Olympus) or confocal microscopy (SPINSR, Olympus).

### 2.13 Transmission Electron Microscopy (TEM)

Fresh hippocampal tissue blocks around 1 mm^3^ were fixed in electron microscopy fixative (Biossci, #BP0130) and post‐fixed with 1% osmium tetroxide (Ted Pella, #02602‐AB). Samples were dehydrated through graded ethanol and acetone, infiltrated, and embedded in SPI‐Pon 812 resin (SPI, #02660‐AB). Ultrathin sections (70 nm) were cut using a Leica UC7 ultramicrotome, double‐stained with uranyl acetate and lead citrate, and imaged using a Hitachi HT7800 transmission electron microscope.

### 2.14 Quantitative Real‐Time PCR (qPCR)

Total RNA was extracted from hippocampal tissues using Trizol reagent (Invitrogen) following standard protocols. cDNA was obtained using the HiScript III RT SuperMix for qPCR (Vazyme, R323‐01). Subsequently, qPCR was performed using ChamQ Universal SYBR qPCR Master Mix (Vazyme, Q711‐02) on a Real‐Time PCR System (Quantstudio3, Thermo Fisher).

### 2.15 Tubulin Polymerization Assay

The effect of TP2 on Tau–induced microtubule assembly was assessed by a turbidity‐based polymerization assay. Reactions were assembled on ice in buffer composed of 80 mm PIPES (pH 6.9), 1 mm EGTA, 2 mm MgCl_2_, and 1 mm GTP. Commercially purified porcine brain tubulin (Cytoskeleton Inc., #T240‐A) was combined with the indicated components: 10 µm tubulin with 5 µm GST‐Tau, 20 µm TP2, or both. Reactions were transferred to a pre‐warmed 96‐well plate, and microtubule assembly was monitored as the change in turbidity (absorbance at 340 nm) at 37°C using a microplate reader (Synergy H1, BioTek), with readings recorded every 3 min over the assembly time course.

### 2.16 Comparison of TP2 and the Peptide‐Based DEPTAC D16

The peptide‐based DEPTAC D16, previously reported by our group [18], was used as a comparator and prepared as a DMSO stock solution. For the concentration‐gradient experiment, primary rat hippocampal neurons (DIV 10–12) were treated with TP2 or D16 at 0, 5, 10, and 20 µm for 24 h. For the washout experiment, neurons were treated with 5 µm TP2 or 20 µm D16 for 24 h, after which the medium was replaced with fresh drug‐free medium; lysates were then collected at 1, 3, 5, and 7 days after washout, with vehicle‐treated neurons included as a control. p‐Tau (AT8), total Tau (Tau5), and β‐actin were detected by Western blotting as described in Section 2.9.

### 2.17 Analysis of TP2‐Induced Tau Degradation

All assays were performed in HEK293‐Tau cells stably expressing human full‐length Tau.

For the cycloheximide (CHX) chase assay, cells were treated simultaneously with 100 µg/mL CHX (MCE, #HY‐12320) and either vehicle or 10 µm TP2 at the start of the chase (t = 0). Cells were harvested at 0, 6, 12, and 24 h, and the levels of AT8, Tau5, and β‐actin were determined by Western blotting. Band intensities were normalized to β‐actin and expressed relative to the 0 h time point.

To identify the degradation pathway, cells were co‐treated with 10 µm TP2 and either the proteasome inhibitor MG132 (10 µm, MCE, #HY‐13259) or the autophagy–lysosome inhibitor bafilomycin A1 (BafA1, 100 nM, MCE, #HY‐100558) for 8 h, and cell lysates were subjected to Western blotting for AT8, Tau5, and β‐actin.

For the Tau ubiquitination assay, cells were treated with 10 µm TP2 for 4 h, after which 10 µm MG132 was added for an additional 4 h to stabilize ubiquitinated species. Cells were lysed under denaturing conditions (boiled in buffer containing 1% SDS and diluted 10‐fold prior to immunoprecipitation). Lysates were incubated with Tau5 antibody (Santa Cruz, #sc‐58860, 1:50) overnight at 4°C, followed by incubation with Protein A/G magnetic beads. Immune complexes were washed, eluted with SDS sample buffer, and immunoblotted with an anti‐Ubiquitin Rabbit mAb (ABclonal, #A19686, 1:500) and Tau5.

### 2.18 Quantification of TP2 in Plasma and Brain by LC–MS/MS

Eight‐week‐old male C57BL/6J mice received a single intravenous (tail vein) injection of vehicle or TP2 (2.5 mg/kg). Thirty minutes after dosing, blood was collected and plasma was separated; mice were then thoroughly perfused to remove residual blood from the cerebral vasculature, and brains were harvested. Brain tissue was homogenized in aqueous methanol (methanol:water, 50:50, v/v) at a ratio of 1 g tissue per 1 mL. For sample preparation, 20 µL of internal standard (IS) working solution and 160 µL of acetonitrile:methanol (1:1, v/v) were added to 20 µL of each calibration, quality‐control (QC), or unknown sample (blank samples received 20 µL of 50% aqueous methanol and 160 µL of acetonitrile). Samples were vortexed for 2 min and centrifuged at 16 000 × g for 5 min at 4°C; 100 µL of the supernatant was mixed with 100 µL of water for analysis.

TP2 and the IS (glibenclamide) were quantified by LC–MS/MS in positive‐ion electrospray (ESI+) multiple‐reaction‐monitoring (MRM) mode, monitoring the transitions m/z 806.2 → 419.0 for TP2 and m/z 494.2 → 369.2 for glibenclamide. Separation was performed on a Waters ACQUITY UPLC HSS T3 column (2.1 × 50 mm, 1.8 µm) with mobile phase A (water containing 0.1% formic acid and 2 mm ammonium acetate) and mobile phase B (acetonitrile) at 0.55 mL/min (injection volume, 3 µL). The gradient (% B) was held at 35% to 0.3 min, increased to 95% by 1.5 min, maintained at 95% until 2.5 min, and returned to 35% at 2.51 min, with a total run time of 3.5 min. The method was linear over 1–1000 ng/mL, and QC samples (3, 50, and 800 ng/mL) showed accuracies within ±15% (89.3%–108.7%). Concentrations in plasma are expressed as ng/mL and in brain as ng/g.

### 2.19 Statistical Analysis

Data are presented as mean ± SEM. ImageJ and GraphPad Prism were used for analysis and visualization. The schematic illustrations were created with BioRender.com Student's *t*‐test was applied for two‐group comparisons, and one‐ or two‐way ANOVA for multiple groups. Significance was defined as *p* < 0.05.

## 3 Results

### 3.1 Rational Design and Chemical Synthesis of TP2

**FIGURE 1:**
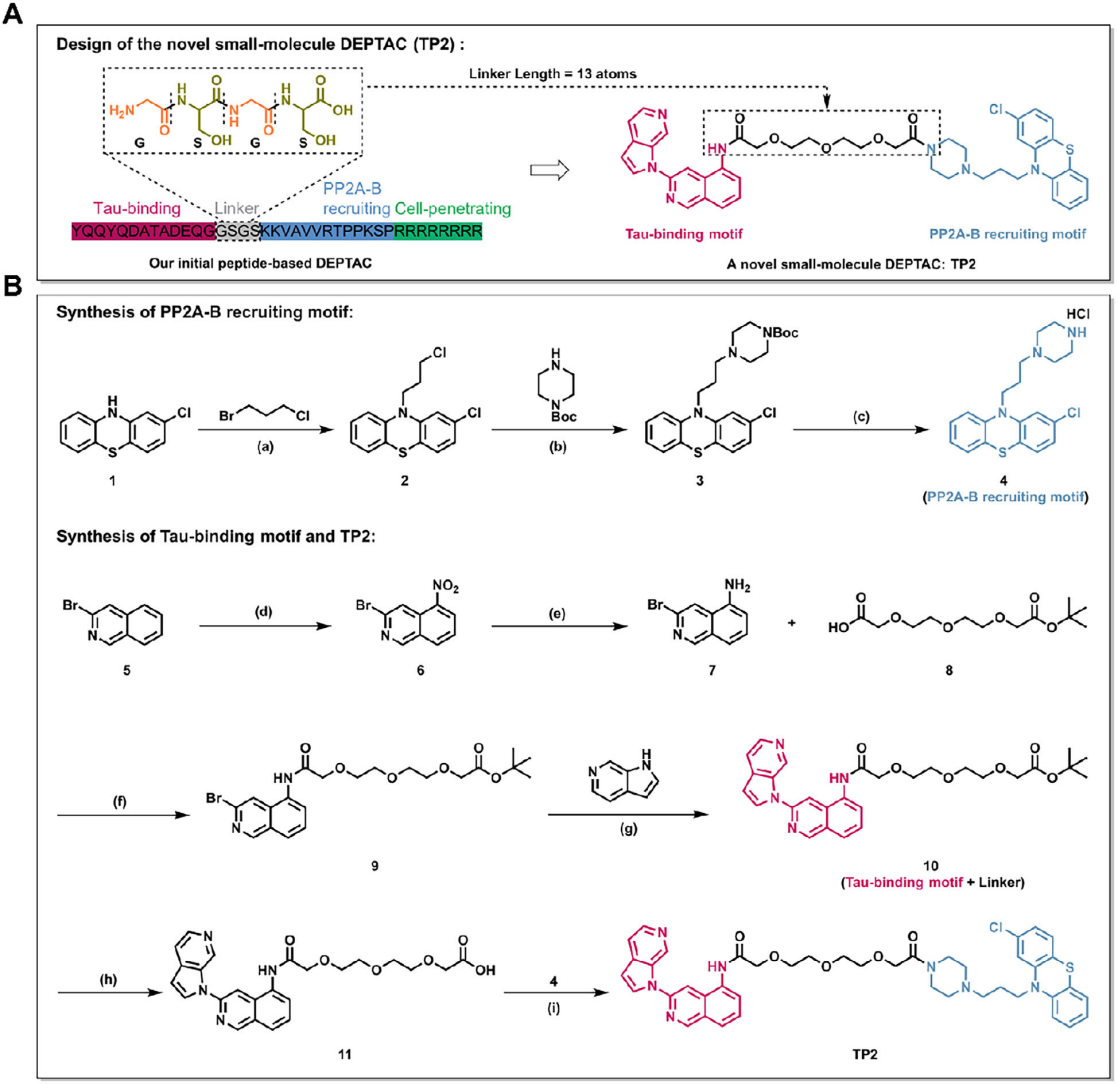
Design and synthesis for TP2. (A) Schematic illustration of the structural evolution from the initial peptide‐based DEPTAC to the small‐molecule TP2. The design incorporates a Tau‐binding motif derived from the high‐affinity pyrrolo[2,3‐c]pyridine derivative (Compound 11–5) and a PP2A‐recruiting motif based on the phenothiazine scaffold (Perphenazine), connected via a PEG‐based linker to maintain optimal spatial distance. (B) The convergent synthetic route for TP2. Reagents and conditions: (a) NaH, THF, N_2_, 65°C, 5 h; (b) TEA, CH_3_CN, 80°C, 4 h; (c) HCl ethyl acetate solution, 0°C, 0.5 h; (d) KNO_3_, Conc. H_2_SO_4_, 0°C to r.t. for 3.5 h; (e) Fe, AcOH, r.t. for 2 h; (f) HATU, DIPEA, DMF, 2 h; (g) Cu_2_O, BFMO, K_3_PO_4_, DMSO, N_2_, 120°C, overnight; (h) TFA, DCM, 0°C to r.t. for 3 h; (i) EDCI, HOBt, DIPEA, DCM, 0°C to r.t. for 2.5 h.

Guided by the design principles of DEPTACs [15, 18, 19, 20], we transitioned our initial peptide‐based DEPTAC into small‐molecule analogs. Based on data from the BindingDB database, the pyrrolo[2,3‐c]pyridine derivative (compound **11–5**) from US Patent US10022461 was identified for its superior binding affinity toward Tau [26]. Consequently, compound **11–5** was selected as the Tau‐binding motif, utilizing its free amino group as the chemical handle for linker attachment. To preserve the recruitment potency toward PP2A, we employed phenothiazine‐based PP2A activators (such as Perphenazine), which have been extensively validated in previous studies [27]. Given that the phenothiazine scaffold resides within the binding pocket of PP2A, the linker was conjugated to the terminal amine of the piperazine ring of Perphenazine. Regarding linker design, a PEG‐based spacer was utilized to bridge the Tau‐binding and PP2A‐Bα‐recruiting motifs, with its length being consistent with that of the peptide‐based DEPTAC (Figure 1A).

The target bifunctional molecule **TP2** was synthesized via a convergent strategy, involving the preparation of a PP2A‐recruiting motif and a Tau‐binding motif, followed by a final amide coupling (Figure 1B). Initially, the PP2A‐recruiting motif **4** was prepared starting from 2‐chlorophenothiazine (**1**). Nucleophilic *N*‐alkylation of **1** with 1‐bromo‐3‐chloropropane in the presence of NaH afforded intermediate **2**, which subsequently underwent nucleophilic substitution with *N*‐Boc‐piperazine to yield **3**. Deprotection of the Boc group using HCl ethyl acetate solution provided the piperazine‐functionalized fragment **4** as a hydrochloride salt. In Parallel, the Tau‐binding motif was constructed starting from 3‐bromoisoquinoline (**5**). Regioselective nitration using KNO_3_ in concentrated H_2_SO_4_ yielded intermediate **6**, which was then reduced by iron powder in acetic acid to give the amino‐substituted isoquinoline **7**. The linker was introduced through an amide coupling between **7** and the carboxylic acid **8** using HATU/DIPEA to afford **9**. A subsequent Copper‐catalyzed Ullmann‐type C‐N coupling between **9** and 6‐azaindole, mediated by Cu_2_O and the BFMO ligand, successfully installed the azaindole moiety to give **10**. Finally, the *tert*‐butyl ester in **10** was removed with TFA to reveal the carboxylic acid **11**. The synthesis was completed by the EDCI/HOBt‐mediated amide bond formation between the Tau‐binding fragment **11** and the PP2A‐recruiting fragment **4**, successfully affording the final product **TP2**.

### 3.2 TP2 Directly Binds Tau and PP2A‐Bα to Induce Ternary Complex Formation

**FIGURE 2:**
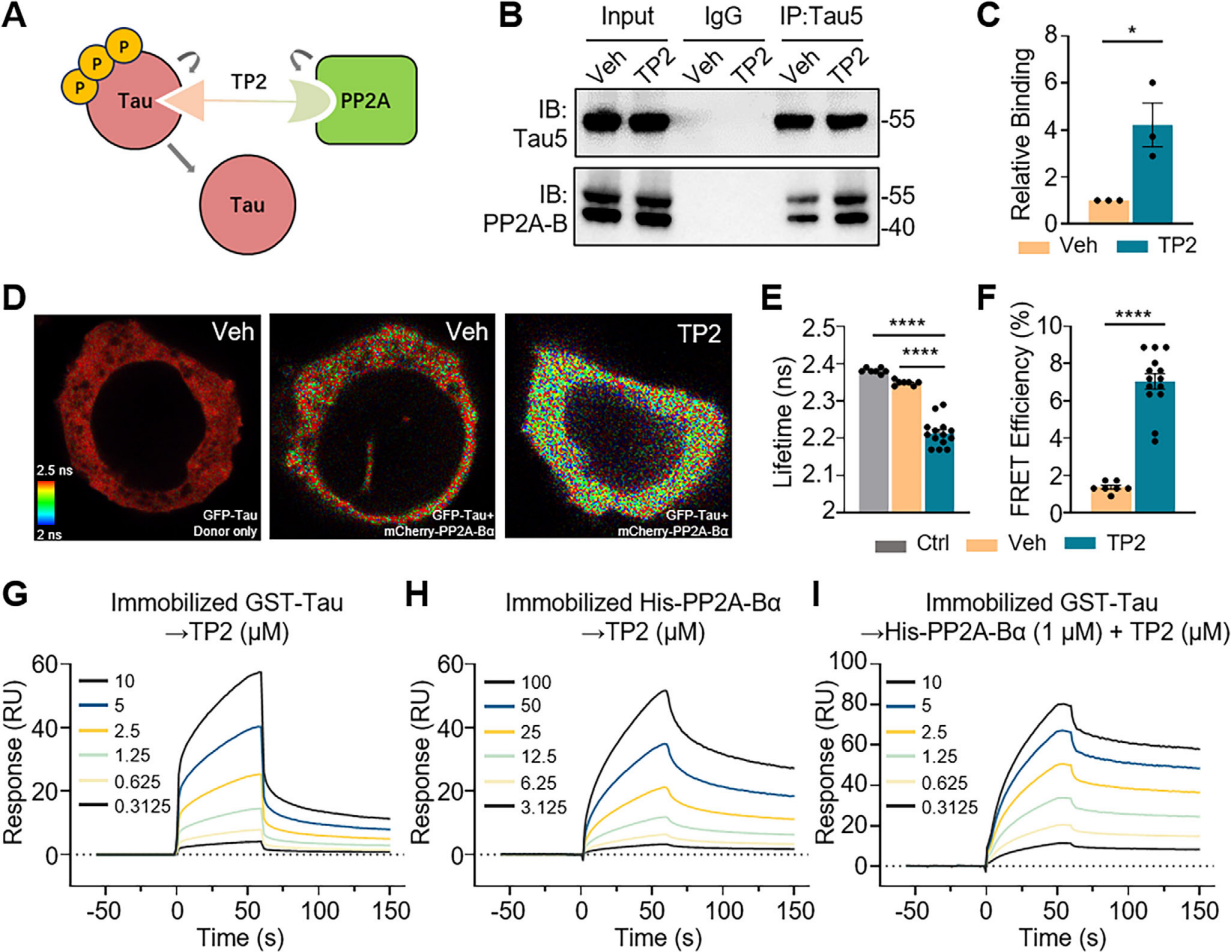
TP2 directly binds Tau and PP2A‐Bα to facilitate their interaction. (A) Schematic illustration of the TP2 design principle. TP2 acts as a molecular bridge, tethering the PP2A‐Bα subunit to Tau to facilitate targeted dephosphorylation. (B, C) The interaction between endogenous Tau and PP2A‐Bα was assessed by Co‐IP. Lysates from primary neurons treated with 5 µm TP2 for 6 h or vehicle were immunoprecipitated with Tau5 antibody and immunoblotted by PP2A B subunit antibody (B). Quantification of the relative binding intensity (C) showed that TP2 treatment enhanced the co‐precipitation of both the PP2A‐Bα isoform (upper band) and the likely homologous isoforms (lower band) compared to the vehicle group. *n* = 3, unpaired Student's *t*‐test, *p < 0.05. (D‐F) FLIM‐FRET assays were performed in live HEK293 cells to detect TP2‐induced Tau–PP2A‐Bα proximity. (D) Representative fluorescence lifetime heatmaps display cells expressing GFP‐Tau alone (Donor only) or co‐expressing GFP‐Tau and mCherry‐PP2A‐Bα treated with vehicle or 5 µm TP2 for 6 h. (E) Quantification of the donor fluorescence lifetime showed a significant decrease in the TP2 group compared to the donor‐only and vehicle controls. (F) Quantification of FRET efficiency demonstrated a significant increase following TP2 treatment, confirming induced molecular proximity. *n* = 7 for ctrl, *n* = 14 for veh and TP2, one‐way ANOVA with Tukey's post‐hoc test for (E) and unpaired Student's *t*‐test for (F). ***p < 0.001, ****p < 0.0001. Data are presented as mean ± SEM. (G–I) Direct binding of TP2 to its targets and TP2‐induced ternary complex formation were characterized by surface plasmon resonance (SPR); sensorgrams are shown with 1:1 Langmuir global fits. (G) TP2 (0.3125–10 µm) injected over immobilized GST‐Tau (*K_D_* = 7.36 µm). (H) TP2 (3.125–100 µm) injected over immobilized His‐PP2A‐Bα (*K_D_* = 92.5 µm). (I) TP2 (0.3125–10 µm) mixed with 1 µm His‐PP2A‐Bα and injected over immobilized GST‐Tau; the presence of PP2A‐Bα increased the apparent affinity of TP2 for Tau (*K_D_* = 2.44 µm), indicating cooperative ternary complex formation.

With the synthesized molecule in hand, we first characterized its ability to function as a molecular bridge between Tau and PP2A (Figure 2A).

To verify that TP2 induces the formation of a ternary complex in a cellular context, we performed co‐immunoprecipitation experiments. Following treatment with TP2, endogenous Tau was immunoprecipitated using the Tau5 antibody. Immunoblotting for the PP2A regulatory B subunit revealed two distinct bands, consistent with the antibody's ability to recognize the PR55α isoform (∼55 kDa) and potentially cross‐react with highly homologous isoforms (such as β or δ). Notably, TP2 treatment led to a significant enrichment of both bands in the Tau immune complex (Figure 2B,C). This suggests that TP2 likely targets a conserved structural interface shared by PR55 family members, thereby effectively recruiting both the predominant Bα isoform and other homologous B‐subunits to the Tau complex.

We further employed Fluorescence Lifetime Imaging Microscopy with Förster Resonance Energy Transfer (FLIM‐FRET) to visualize this proximity induction in living cells. Cells co‐expressing GFP‐Tau (FRET donor) and mCherry‐PP2A‐Bα (FRET acceptor) were treated with TP2. As shown in the representative heatmaps (Figure 2D), TP2 treatment resulted in a significant decrease in the fluorescence lifetime of the GFP‐Tau donor (Figure 2E) and a corresponding robust increase in FRET efficiency (Figure 2F). This indicates that TP2 successfully brings Tau and PP2A‐Bα into close molecular proximity (<10 nm).

Finally, to obtain direct evidence of target engagement and complex formation, we turned to surface plasmon resonance (Table 1). TP2 bound directly to immobilized GST‐Tau with a dissociation constant (*K_D_*) of 7.36 µm and to His‐PP2A‐Bα with a *K_D_* of 92.5 µm, confirming engagement of both proteins with a markedly higher affinity for Tau (Figure 2G,H). Importantly, this engagement did not compromise the physiological function of Tau: in a tubulin turbidity assay, TP2 alone did not promote microtubule assembly and did not alter Tau‐induced assembly across any kinetic parameter, indicating that TP2 binding leaves Tau's microtubule‐stabilizing activity intact (Figure S1). Most notably, when TP2 was mixed with 1 µm PP2A‐Bα and injected over immobilized GST‐Tau, its apparent affinity for Tau increased approximately three‐fold to a *K_D_* of 2.44 µm, demonstrating that Tau, TP2, and PP2A‐Bα assemble into a cooperative ternary complex in which PP2A‐Bα stabilizes the TP2–Tau interaction (Figure 2I).

**TABLE 1:** SPR‐derived binding kinetics and affinities of TP2 for immobilized Tau and PP2A‐Bα.

Immobilized protein	Injected analyte	*K_D_* (µm)	*k_a_* (M^−1^ s^−1^)	*k_d_* (s^−1^)
GST‐Tau	TP2	7.36	1.22 × 10^4^	8.97 × 10^−^^2^
His‐PP2A‐Bα	TP2	92.5	2.66 × 10^3^	2.46 × 10^−^^1^
GST‐Tau	His‐PP2A‐Bα (1 µm) + TP2	2.44	3.80 × 10^3^	9.26 × 10^−^^3^

Collectively, these endogenous co‐immunoprecipitation, live‐cell imaging, and biophysical data provide multi‐level validation that TP2 functions as a molecular bridge that physically tethers PP2A‐Bα to Tau through a cooperative ternary complex.

### 3.3 TP2 Promotes Potent Tau Dephosphorylation and Rescues Tau‐Mediated Neurotoxicity in Primary Neurons

**FIGURE 3:**
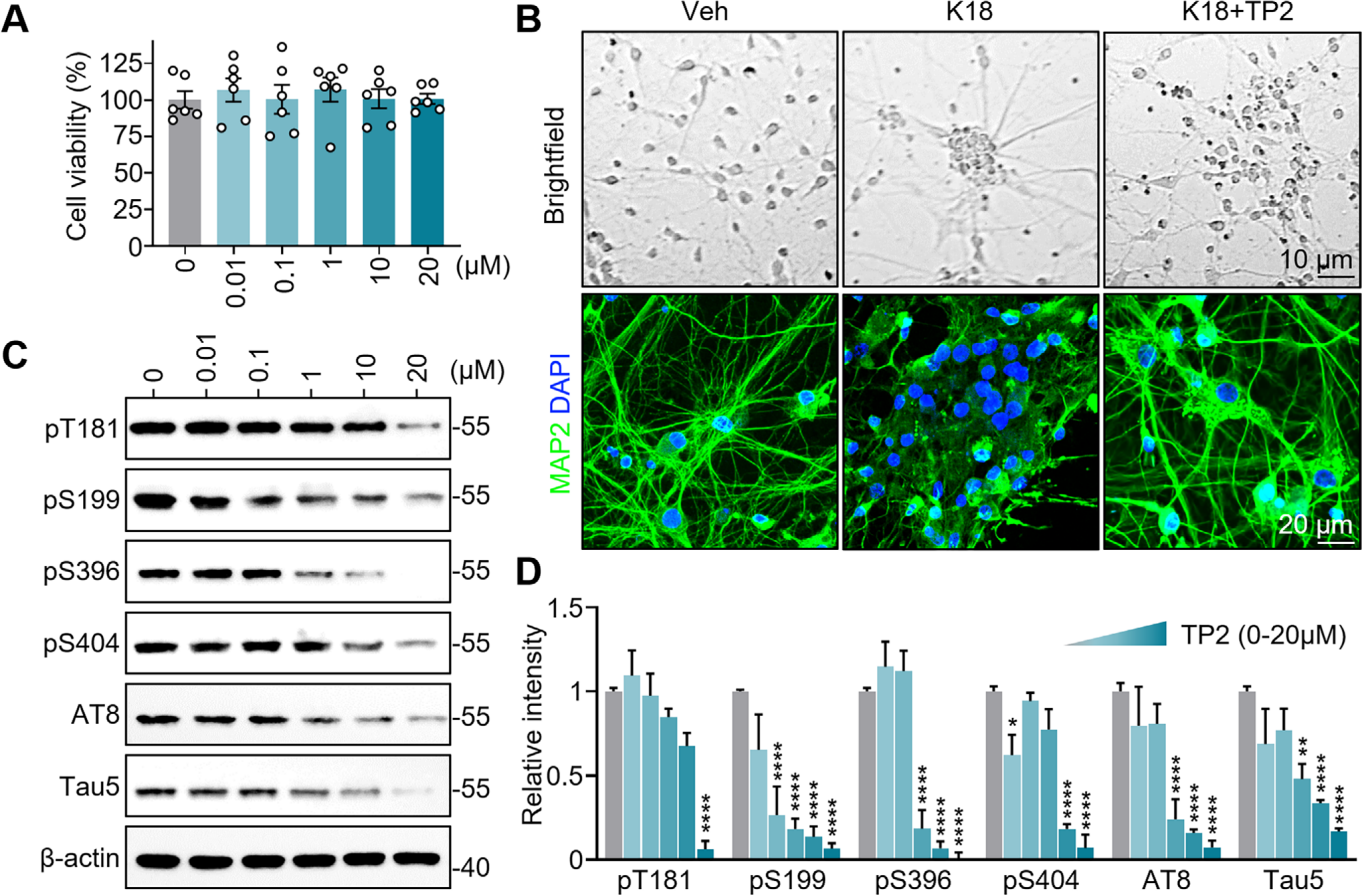
TP2 effectively reduces Tau phosphorylation and rescues K18‐induced neurotoxicity in primary neurons. (A) CCK‐8 assays were performed on primary rat hippocampal neurons treated with increasing concentrations of TP2 (0–20 µm) for 24 h. Quantification of cell viability indicated no significant difference across the tested concentrations, suggesting no cytotoxicity. *n* = 6, one‐way ANOVA. (B) Bright‐field imaging and immunofluorescence staining for MAP2 (green) counterstained with DAPI (blue) were conducted to assess neuronal morphology. Representative images show that K18 fibrils induced somatic aggregation and neurite dystrophy compared to the vehicle group, while TP2 treatment effectively ameliorated these pathological changes. (C, D) Western blotting was performed to detect Tau levels in primary neurons treated with different concentrations of TP2 (0–20 µm) for 24 h. Representative blots (C) and quantification of the relative intensity normalized to β‐actin (D) show that TP2 treatment dose‐dependently reduced the levels of multiple phosphorylated Tau epitopes (pT181, pS199, pS396, pS404, AT8) and total Tau (Tau5). *n* = 3, one‐way ANOVA with Dunnett's post‐hoc test, *p < 0.05, **p < 0.01, ****p < 0.0001 vs. 0 µm. Data are presented as mean ± SEM.

Having established that TP2 can physically bridge Tau and PP2A, we next investigated whether this recruitment functionally promotes the dephosphorylation of Tau within neurons. Treatment of primary rat hippocampal neurons with TP2 resulted in a potent and dose‐dependent reduction in Tau phosphorylation across multiple epitopes, including pT181, pS199, pS396, pS404 and AT8 (pS202/T205), accompanied by a concomitant decrease in total Tau (Tau5) (Figure 3C,D). Crucially, CCK‐8 assay confirmed that TP2 did not impact neuronal viability across the full range of effective concentrations (Figure 3A).

To compare the performance of this fully synthetic small‐molecule DEPTAC with that of a previously reported peptide‐based DEPTAC operating through the same PP2A‐Bα recruitment principle, we evaluated TP2 alongside D16 [18]. TP2 achieved comparable Tau dephosphorylation at lower effective concentrations and maintained a more sustained reduction in Tau phosphorylation after drug washout, whereas total Tau levels remained comparable between the two treatment groups during the washout period (Figure S2). These findings demonstrate greater cellular potency and more sustained dephosphorylation by TP2.

To clarify the reduction in total Tau induced by TP2 treatment, we performed a CHX‐chase assay in HEK293‐Tau cells and found that TP2 accelerated Tau degradation (Figure S3A,C–E). MG132, but not bafilomycin A1, prevented the TP2‐associated decrease in Tau5, indicating that this reduction occurs predominantly through the proteasome pathway (Figure S3B,F–H). Consistently, TP2 increased Tau ubiquitination (Figure S3I,J). Notably, MG132 restored Tau5 without reversing the reduction in AT8, indicating that TP2‐induced dephosphorylation precedes the subsequent proteasome‐dependent reduction in total Tau. Together, these findings indicate that TP2 primarily promotes Tau dephosphorylation, after which Tau becomes more susceptible to ubiquitin–proteasome‐dependent degradation, consistent with previous evidence [28].

We then sought to determine if TP2's potent dephosphorylating activity could protect neurons from a direct, tau‐mediated toxic insult. To model this, we utilized exogenous fibrils formed from K18, the aggregation‐prone microtubule‐binding repeat domain of Tau. This model is well‐established to effectively seed the aggregation and subsequent hyperphosphorylation of endogenous Tau, thereby recapitulating key aspects of tauopathy progression in vitro [18, 29, 30]. As expected, exposure of primary neurons to K18 fibrils induced pronounced pathological changes, including somatic aggregation and severe neurite dystrophy, as visualized by bright‐field microscopy and MAP2 immunofluorescence (Figure 3B). Remarkably, TP2 treatment ameliorated this neurotoxic phenotype, preserving a healthier neuronal morphology and maintaining the integrity of the dendritic network despite the presence of toxic K18 fibrils.

Together, these results demonstrate that TP2 not only effectively reduces Tau phosphorylation in primary neurons without compromising their viability, but also rescues them from Tau‐driven neurotoxicity, highlighting its therapeutic potential at the cellular level.

### 3.4 Systemic Administration of TP2 Reverses the Full Spectrum of Tau Pathology in Tauopathy Models

To determine whether systemically administered TP2 reaches brain tissue, we quantified its concentrations in plasma and brain by LC–MS/MS following a single intravenous (i.v.) dose of TP2 (2.5 mg/kg) or vehicle in wild‐type C57BL/6J mice. Thirty minutes after administration, TP2 was reliably detected in brain homogenates obtained after thorough perfusion, with a mean concentration of 4.33 ± 0.34 ng/g and a brain‐to‐plasma ratio of approximately 0.034. These data provide direct evidence of brain exposure following systemic administration of TP2 (Figure S4 and Table S1). Considering blood–brain barrier (BBB) dysfunction observed in aged rTg4510 mice, we used wild‐type C57BL/6J mice in the present study to minimize any potential confounding by tauopathy‐associated alterations in BBB permeability [31]. Furthermore, previous brain‐distribution studies of CNS drug candidates were also conducted in C57BL/6J mice [32, 33].

The therapeutic potential of TP2 was assessed in male P301L and AAV‐hTau‐N368 mice following a systemic, multi‐dose treatment schedule. In the P301L cohort, TP2 or vehicle treatment was initiated at 12 months of age. For the AAV‐hTau‐N368 cohort, 8‐week‐old male C57BL/6J mice received bilateral stereotaxic injections of AAV‐SYN‐EGFP‐2A‐hTau‐N368 into the hippocampal dentate gyrus; after 4 weeks of viral expression, TP2 or vehicle treatment was initiated when the mice were 3 months old. Mice in both models received TP2 (2.5 mg/kg) or vehicle through intravenous (i.v.) tail‐vein injection every 4 days for a total of seven doses, and behavioral testing was initiated 2 days after the final administration.

**FIGURE 4:**
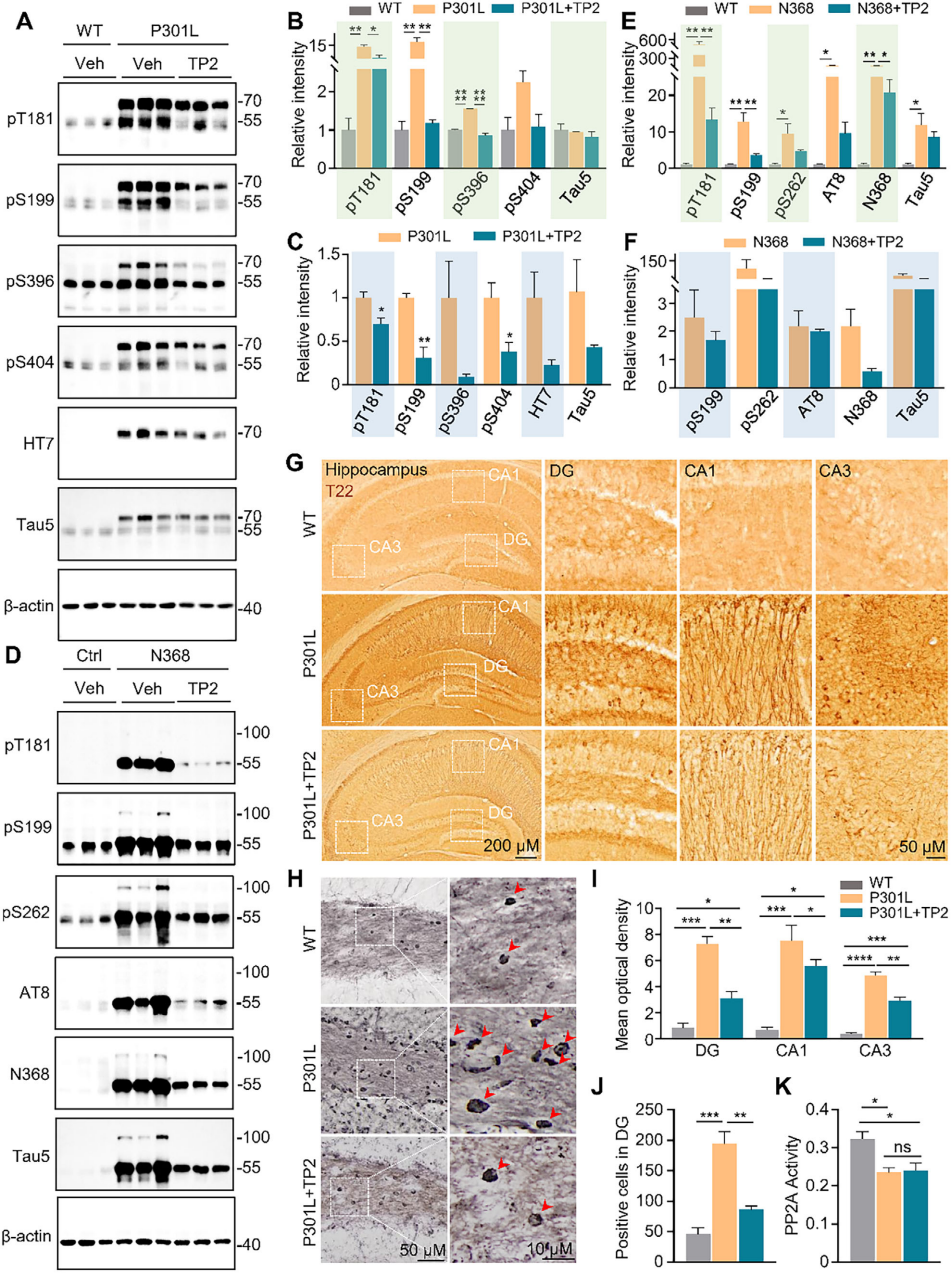
TP2 effectively reduces hyperphosphorylated, oligomeric, and aggregated Tau in tauopathy models without altering global PP2A activity. (A–C) Western blotting was performed on hippocampal lysates from WT and 12‐month‐old P301L mice treated with vehicle or TP2 to assess Tau pathology. Representative blots (A) and quantification of the relative band intensity normalized to β‐actin (B, C) reveal that TP2 treatment reduced the phosphorylation of both endogenous mouse Tau (∼55 kDa, quantified in B) and exogenous human P301L Tau (∼70 kDa, quantified in C), whereas total Tau showed only a modest, non‐significant downward trend. *n* = 3, one‐way ANOVA with Tukey's post‐hoc test for (B), unpaired Student's *t*‐test for (C). *p < 0.05, **p < 0.01, **** *p* < 0.0001. (D–F) Western blotting was performed on hippocampal lysates from control and AAV‐hTau‐N368 mice treated with vehicle or TP2. Representative blots (D) and quantification of the relative band intensity normalized to β‐actin (E, F) reveal that TP2 treatment decreased the phosphorylation of Tau (∼55 kDa, quantified in E) and the high molecular weight aggregated Tau (∼100 kDa, quantified in F). *n* = 3, one‐way ANOVA with Tukey's post‐hoc test for (E), unpaired Student's *t*‐test for (F). *p < 0.05, **p < 0.01. (G, I) Immunohistochemical staining with the T22 antibody was used to visualize oligomeric Tau in the hippocampus. Representative images (G) and quantification of the mean optical density (I) indicate that TP2 treatment significantly lowered the levels of Tau oligomers compared to the vehicle‐treated P301L group. *n* = 3, one‐way ANOVA with Tukey's post‐hoc test, *p < 0.05, **p < 0.01, ***p < 0.001. (H, J) Gallyas silver staining was performed to detect neurofibrillary tangles in the dentate gyrus (DG). Representative images (H) and quantification of the number of positive cells (J) show a significant reduction in silver‐positive aggregates following TP2 treatment. *n* = 3, one‐way ANOVA with Tukey's post‐hoc test, **p < 0.01, ***p < 0.001. (K) PP2A phosphatase activity assay was carried out on hippocampal lysates to evaluate enzyme function. Quantification of relative PP2A activity showed that while P301L mice exhibited reduced phosphatase activity compared to WT mice, TP2 treatment did not alter the global PP2A activity in P301L mice compared to the vehicle group. *n* = 3, one‐way ANOVA with Tukey's post‐hoc test, *p < 0.05. Data were presented as mean ± SEM.

In P301L transgenic mice that began treatment at 12 months of age, Western blot analysis of hippocampal lysates revealed that TP2 treatment induced a widespread reduction in multiple Tau phosphorylation epitopes (Figure 4A–C). Notably, in contrast to the profound suppression of phosphorylated Tau, total Tau levels (detected by HT7, especially targeting human Tau, and Tau5) exhibited only a modest decreasing trend that did not reach statistical significance. This distinct profile highlights the key therapeutic advantage of TP2. Rather than indiscriminately depleting the total Tau pool, TP2 effectively rectifies the pathological Tau phosphorylation imbalance while sparing the protein levels necessary for physiological function. This p‐Tau modulation was consistently observed in cortical lysates in P301L (Figure S5A–C) and further corroborated in the AAV‐mediated hTau‐N368 model, where TP2 similarly ameliorated Tau phosphorylation and neurotoxic N368 while exhibiting a reduced trend regarding the levels of total Tau (Figure 4D–F).

Beyond reducing soluble p‐Tau, we investigated whether TP2 could impact more mature, aggregated forms of tau pathology. Analysis of the insoluble fraction from the hippocampus of P301L mice revealed a widespread decrease in phosphorylated Tau, indicating that TP2 can effectively target Tau even after it has aggregated into less soluble species (Figure S6A–C). This finding was corroborated by immunohistochemical analysis. Staining with the T22 antibody revealed a marked reduction in oligomeric Tau, a species widely considered to be a primary driver of neurotoxicity [34, 35], throughout the hippocampal subfields of TP2‐treated P301L mice (Figure 4G,I). Furthermore, Gallyas silver staining, which labels dense‐core neurofibrillary tangles (NFTs), demonstrated a significant decrease in the number of NFT‐positive neurons in the dentate gyrus of mice that received TP2 treatment (Figure 4H,J). The capacity of TP2 to ameliorate the full spectrum of Tau pathology, from nascent hyperphosphorylation to insoluble aggregates and mature tangles, highlights the therapeutic advantage of targeting the upstream post‐translational modification.

A central tenet of the DEPTAC mechanism is the targeted recruitment of phosphatases without altering their global enzymatic activity. To confirm this specific mode of action in vivo, we performed a PP2A phosphatase activity assay on hippocampal lysates. As previously reported, P301L mice exhibited a significant reduction in basal PP2A activity [24]. Crucially, treatment with TP2 did not alter the global PP2A activity level (Figure 4K), confirming that TP2 acts as a true molecular recruiter rather than a general phosphatase activator.

### 3.5 TP2 Rebuilds Neuronal Architecture and Restores Synaptic Integrity

**FIGURE 5:**
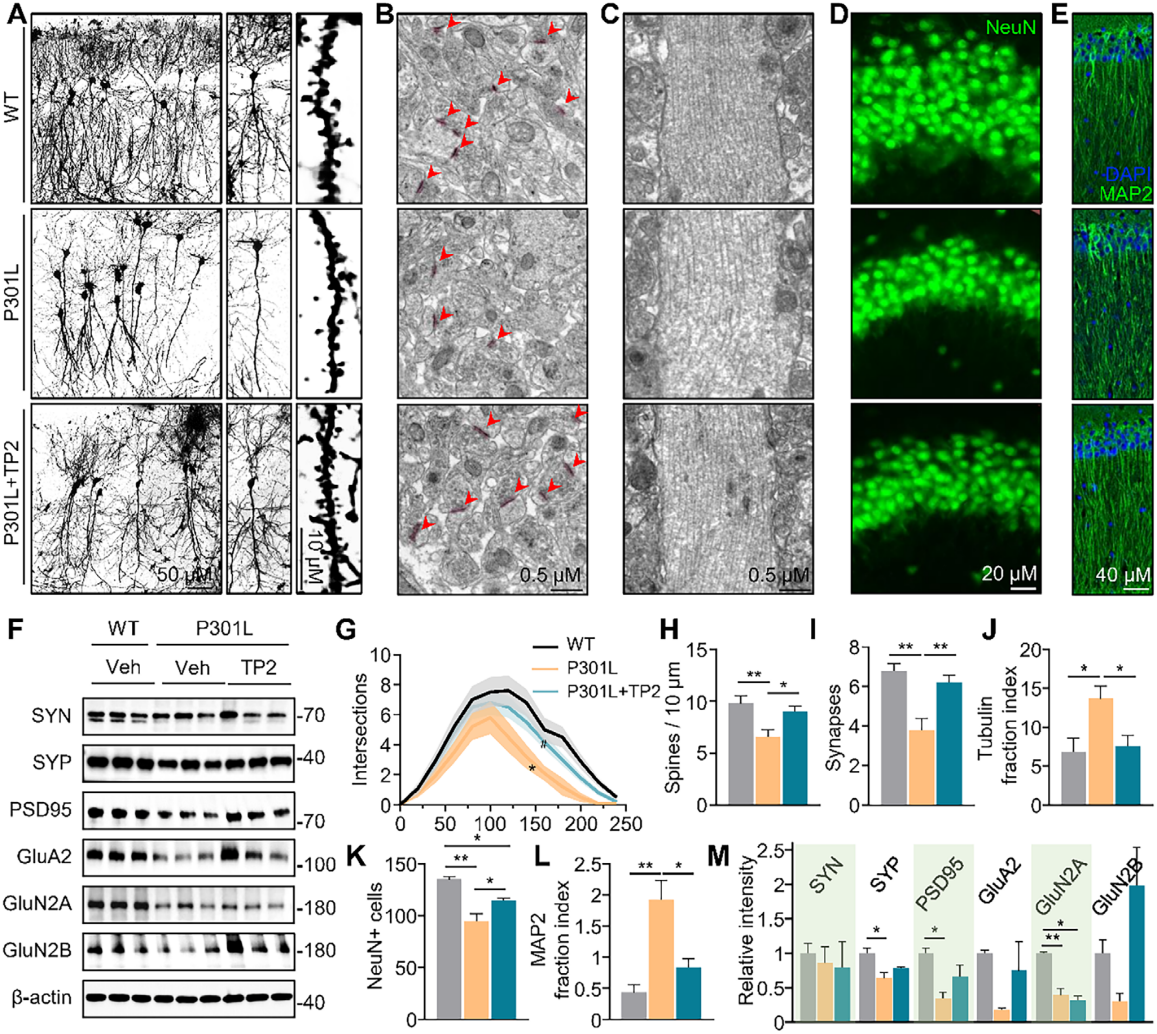
TP2 treatment rescues neuronal morphology, synaptic integrity, and cytoskeletal structure in P301L mice. (A, G, H) Golgi‐Cox staining was performed to visualize the dendritic morphology of CA1 pyramidal neurons. Representative images (A), Sholl analysis of dendritic intersections (G), and quantification of spine density (H) show that TP2 treatment restored both dendritic complexity and spine density compared to the P301L group. Two‐way repeated measures ANOVA (for Sholl analysis) or one‐way ANOVA (for spine density), Tukey's post‐hoc test. In (G), *p < 0.05 vs. WT, #*p* < 0.05 vs. P301L. In (H), *p < 0.05, ** *p* < 0.01. *n* = 10. (B, I) Transmission electron microscopy (TEM) was performed to detect electron‐dense synaptic active zones. Representative images (B) and quantification (I) reveal that TP2 treatment increased the number of synaptic active zones (pseudocolored in red, arrowheaded). One‐way ANOVA with Tukey's post‐hoc test, **p < 0.01. *n* = 5 (C, J) TEM was performed to examine microtubule ultrastructure. Representative images of microtubules (C) and quantification of the tubulin fraction index (J) show that TP2 treatment reduced microtubule fragmentation. One‐way ANOVA with Tukey's post‐hoc test, *p < 0.05. *n* = 10. (D, K) Immunofluorescence staining for NeuN was conducted to assess neuronal number in the CA3 region. Representative images (D) and quantification of NeuN‐positive cells (K) show that TP2 treatment reversed neuronal loss found in P301L mice. One‐way ANOVA with Tukey's post‐hoc test, *p < 0.05, **p < 0.01. *n* = 3. (E, L) Immunofluorescence staining for MAP2 (green) counterstained with DAPI (blue) was performed to evaluate dendritic integrity in the CA1 region. Representative images (E) and quantification of the MAP2 fraction index (L) show that TP2 treatment preserved dendritic structural integrity. One‐way ANOVA with Tukey's post‐hoc test, *p < 0.05, **p < 0.01. *n* = 3. (F, M) Western blotting was carried out to measure the expression of synaptic proteins. Representative blots (F) and quantification of relative intensity normalized to β‐actin (M) indicate that TP2 treatment partially restored the levels of presynaptic and postsynaptic protein. One‐way ANOVA with Tukey's post‐hoc test, *p < 0.05. *n* = 3. Data were presented as mean ± SEM.

We next assessed the impact of TP2 on the structural hallmarks of neurodegeneration in P301L mice. In the hippocampal CA3 region, TP2 treatment reversed the significant neuronal loss observed in vehicle‐treated P301L mice, as quantified by NeuN immunofluorescence (Figure 5D,K).

Beyond preserving neuron numbers, TP2 treatment also restored the health of the dendritic architecture. Golgi‐Cox staining of CA1 pyramidal neurons revealed that TP2 reversed the severe dendritic atrophy observed in P301L mice, rescuing both dendritic complexity and density of dendritic spines (Figure 5A,G,H). The preservation of this intricate dendritic network was further corroborated by MAP2 immunofluorescence, which demonstrated a recovery of dendritic integrity in the CA1 region (Figure 5E,L).

This rescue of dendritic architecture was mirrored at ultrastructural and molecular levels. Transmission electron microscopy (TEM) in hippocampus revealed that TP2 treatment reversed the marked loss of synaptic density observed in P301L mice (Figure 5B,I). Consistent with this finding, Western blot analysis of hippocampal lysates indicated a trend toward preserved expression levels of presynaptic SYP and postsynaptic proteins (PSD95, GluA2 and GluN2B) (Figure 5F,M). Finally, to directly assess the restoration of Tau's physiological function, we examined the microtubule cytoskeleton. TEM analysis showed that TP2 reversed the microtubule fragmentation in P301L mice, as quantified by tubulin fraction index (Figure 5C,J).

### 3.6 TP2 Improves Neuroimmune Microenvironment by Attenuating Microglial Activation and Alleviating Astrocytic Atrophy

**FIGURE 6:**
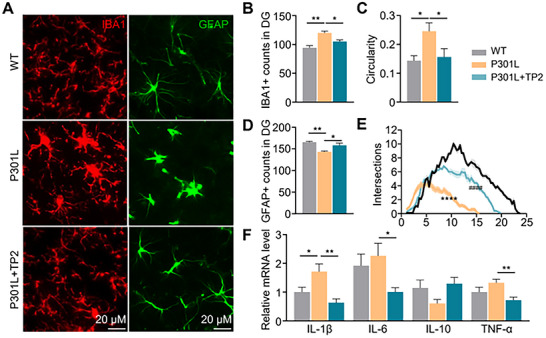
Effects of TP2 treatment on microglial activation and astrocytic atrophy in P301L mice. (A–C) Immunofluorescence staining for IBA1 was performed to assess microglia in the dentate gyrus (DG). Representative images (A) and quantification of the number (B) and circularity (C) of IBA1‐positive cells show that TP2 treatment reduced both the number and circularity of microglia compared to the P301L group. One‐way ANOVA with Tukey's post‐hoc test, *p < 0.05, **p < 0.01. *n* = 3 individual mice. (A, D, E) Immunofluorescence staining for GFAP was performed to assess astrocytes in DG. Representative images (A), quantification of the number of GFAP‐positive astrocytes (D), and Sholl analysis of astrocytic processes (E) show that TP2 treatment increased the number and process complexity of astrocytes compared to the P301L group. In (D), * *p* < 0.05, ** *p* < 0.01. In (E), ****p < 0.0001 vs. WT, ####*p* < 0.0001 vs. P301L. *n* = 3 individual mice. (F) qPCR was carried out to measure the transcription of inflammatory cytokines. Quantification of relative mRNA levels shows that TP2 treatment significantly decreased the levels of IL‐1β, IL‐6, and TNF‐α, whereas IL‐10 levels exhibited an increasing trend that did not reach statistical significance compared to the P301L group. One‐way ANOVA with Tukey's post‐hoc test, *p < 0.05, **p < 0.01. Data were presented as mean ± SEM, *n* = 3 individual mice.

Neuroinflammation, driven by the dysregulation of glial cells, is a critical propagator of neurodegeneration in tauopathies. To determine if TP2 treatment could ameliorate this pathological microenvironment, we examined the status of microglia and astrocytes in the dentate gyrus (DG) of P301L mice. Immunofluorescence staining for the microglial marker IBA1 revealed a pronounced activation phenotype in vehicle‐treated P301L mice, characterized by a significant increase in number and a shift toward an ameboid morphology, as quantified by increased cell circularity (Figure 6A–C). TP2 treatment effectively suppressed this microglial overactivation, significantly reducing the number of IBA1‐positive cells and reverting their morphology to a more quiescent, ramified state.

Concurrent with microglial activation, we observed a distinct impairment in the astrocyte. Vehicle‐treated P301L mice exhibited signs of astrocytic loss and atrophy, evidenced by a significant reduction in the number of GFAP‐positive cells and a marked decrease in process complexity compared to wild‐type controls (Figure 6A,D,E). While reactive astrogliosis is prevalently reported in P301L models, the decreased number and atrophy observed here likely reflects a transition from activation to glial degeneration consequent to the advanced age (12 months) of P301L mice. This observation also aligns with previous reports characterizing astrocytic atrophy in 3xTg‐AD mice and, crucially, recapitulates the glial pathology observed in human AD brains [36, 37]. Strikingly, TP2 treatment reversed this astrocytic dystrophy, restoring both the GFAP+ cell density and the morphological complexity of astrocytes to levels comparable to wild‐type mice (Figure 6D,E).

This restoration of glial homeostasis was further corroborated by qPCR analysis of hippocampal tissue. TP2 treatment significantly downregulated the mRNA expression of key pro‐inflammatory cytokines, including IL‐1β, IL‐6, and TNF‐α, which were elevated in P301L mice. Conversely, TP2 also induced a modest elevation in anti‐inflammatory cytokines IL‐10, although this did not reach statistical significance (Figure 6F).

Collectively, these data suggest that TP2 exerts a dual beneficial effect on the neuroimmune environment: dampening neurotoxic microglial inflammation while rescuing neuroprotective astrocytic integrity.

### 3.7 Systemic Administration of TP2 Reverses Cognitive Deficits in Tauopathy Models

**FIGURE 7:**
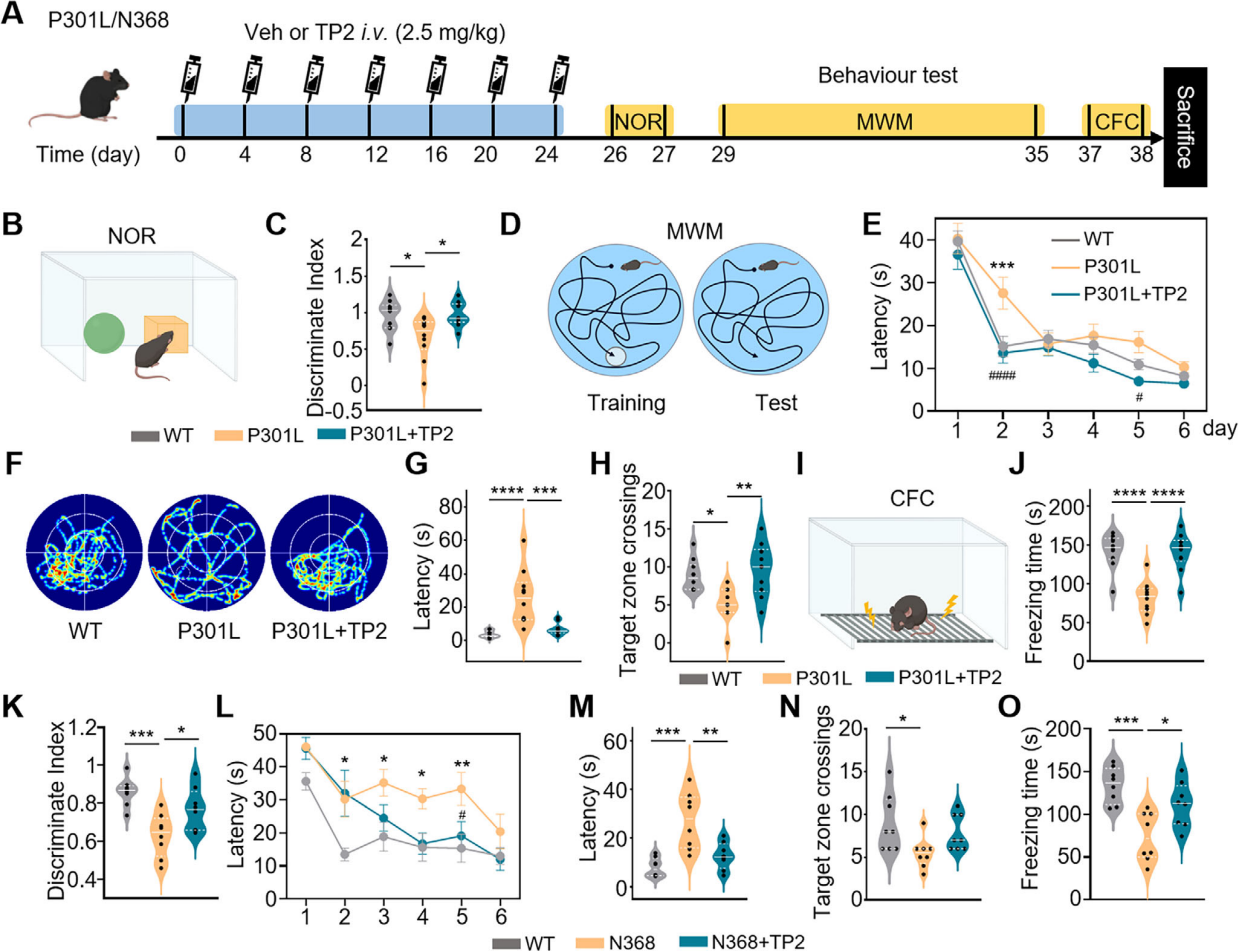
TP2 administration ameliorates cognitive deficits in P301L and AAV‐hTau‐N368 mouse models. (A) Experimental timeline for TP2 administration and behavioral tests. Treatment of P301L mice was initiated at 12 months of age. For the AAV‐hTau‐N368 cohort, 8‐week‐old male C57BL/6J mice received bilateral stereotaxic injections of AAV‐SYN‐EGFP‐2A‐hTau‐N368 into the hippocampal dentate gyrus; TP2 or vehicle treatment was initiated 4 weeks later, when mice were 3 months old. (B, C) TP2 increased the discrimination index of P301L mice in the NOR test. The cartoon (B) illustrates the paradigm of the NOR test. One‐way ANOVA with Tukey's post‐hoc test, *p < 0.05. (D, E) TP2 reduced the escape latency of P301L mice to find the hidden platform during MWM training phase. The cartoon (D) illustrates the paradigm of MWM test. Two‐way repeated measures ANOVA with Tukey's post‐hoc test, ***p < 0.001 vs. WT, ####*p* < 0.0001 vs. P301L. (F–H) TP2 decreased the latency to reach the target zone (G) and increased the number of platform crossings (H) of P301L mice in the MWM probe phase. Representative heatmaps (F) show the search path. One‐way ANOVA with Tukey's post‐hoc test, *p < 0.05, **p < 0.01, ***p < 0.001, **** *p* < 0.0001. (I, J) TP2 increased the freezing time of P301L mice in the CFC test. The cartoon (I) illustrates the paradigm of the CFC test. One‐way ANOVA with Tukey's post‐hoc test, ****p < 0.0001. (K‐O) TP2 increased the discrimination index (K), reduced the escape latency to find the hidden platform during MWM training phase (L), decreased the latency to reach the target zone (M), increased the number of platform crossings (N) in the MWM probe phase, increased freezing time (O) of AAV‐hTau‐N368 mice. Two‐way repeated measures ANOVA (for L) or one‐way ANOVA (for K, M‐O). In (L), *p < 0.05, **p < 0.01 vs. WT, #*p* < 0.05 vs. N368. In (K, M‐O), *p < 0.05, **p < 0.01, ***p < 0.001. For the P301L cohort (B‐J), *n* = 10 mice per group. For the AAV‐hTau‐N368 cohort (K‐O), *n* = 8 for WT, *n* = 9 for AAV‐hTau‐N368 + vehicle, and *n* = 8 for AAV‐hTau‐N368 + TP2 mice per group. Data are presented as mean ± SEM.

Finally, to determine if the comprehensive rescue of Tau pathology, neuronal structure, and neuroimmune environment translates into functional recovery, we evaluated the cognitive performance by a series of behavioral tests. As outlined in the experimental timeline (Figure 7A), mice received systemic TP2 treatment prior to assessment in the Novel Object Recognition (NOR), Morris Water Maze (MWM), and Contextual Fear Conditioning (CFC) tasks.

While vehicle‐treated P301L mice exhibited severe cognitive deficits, TP2 treatment robustly rescued these impairments across multiple cognitive functions. In the NOR test, which assesses recognition memory, TP2‐treated mice displayed a restored preference for the novel object, achieving a discrimination index comparable to that of wild‐type mice (Figure 7B,C). Spatial learning and memory capabilities were evaluated using the MWM task (Figure 7D). During the training phase, vehicle‐treated P301L mice showed a significant learning deficit, evidenced by prolonged escape latencies. Strikingly, TP2 administration improved learning trajectories, significantly reducing the latency to find the platform (Figure 7E). In the subsequent probe trial, TP2‐treated mice demonstrated restored spatial memory retention. They located the target zone more rapidly and crossed the platform location more frequently than vehicle‐treated controls, a recovery visually corroborated by the swim path heatmaps (Figure 7F–H). Furthermore, in the CFC test, TP2 treatment significantly increased freezing time, indicating a successful restoration of fear memory consolidation (Figure 7I,J).

To verify the broad therapeutic potential of TP2, we validated these findings in the AAV‐hTau‐N368 model, which represents a distinct, fragment‐induced tauopathy. Consistent with the results in P301L mice, systemic TP2 treatment significantly ameliorated cognitive deficits in this model as well. TP2‐treated N368 mice showed improved discrimination in the NOR test (Figure 7K), faster learning and better memory retention in the MWM (Figure 7L–N), and robust memory recovery in the CFC test (Figure 7O).

Collectively, these behavioral data provide compelling evidence that TP2 is a potent therapeutic agent capable of reversing cognitive dysfunction in diverse models of tauopathy by targeting the underlying Tau pathology.

## 4 Discussion

Targeting Tau pathology represents a pivotal strategy for disease modification in AD and other tauopathies. In this study, we present TP2, a first‐in‐class, small‐molecule DEPTAC that harnesses the endogenous catalytic power of PP2A to eliminate pathological p‐Tau. Our data provide compelling preclinical evidence that TP2 effectively reverses the core pathological, structural, and functional hallmarks of tauopathy.

A major limitation of kinase inhibitors is their indiscriminate suppression of signaling pathways essential for cell survival. In contrast, TP2 employs a “proximity‐induced dephosphorylation” mechanism. By physically tethering PP2A to Tau, TP2 increases the local concentration of the enzyme specifically at the substrate site, thereby enhancing specificity. Crucially, our in vivo data confirmed that TP2 does not alter global PP2A phosphatase activity, suggesting a favorable safety profile devoid of the toxicity associated with systemic phosphatase activators. Furthermore, unlike ASOs or PROTACs that may deplete the physiological pool of Tau required for microtubule stabilization, TP2 targets the post‐translational modification itself. We observed that TP2 treatment not only reduced p‐Tau but also restored microtubule integrity and dendritic complexity, supporting the hypothesis that dephosphorylation restores Tau's physiological function in stabilizing the cytoskeleton.

By using a cell‐free tubulin polymerization assay, we found that TP2 binding per se did not measurably impair Tau‐mediated microtubule assembly. In cellular experiments, TP2‐mediated dephosphorylation preceded the subsequent ubiquitin–proteasome‐dependent reduction of Tau, consistent with a model in which correction of pathological phosphorylation enhances Tau susceptibility to endogenous proteostatic turnover, rather than TP2 directly forcing Tau degradation. These data suggest that Tau abundance and Tau function are not necessarily equivalent. Elevated Tau phosphorylation compromises its microtubule binding and bundling, thereby disrupting its physiological role in microtubule stabilization, and can also reduce its susceptibility to proteasomal degradation [38, 39]. Accordingly, the modest reduction in total Tau following correction of its pathological phosphorylation state should not be interpreted as a loss of physiological Tau function. This apparent dissociation between Tau abundance and restored microtubule integrity instead highlights a key advantage of correcting pathological phosphorylation rather than indiscriminately depleting Tau.

Our study also sheds light on the complex interplay between Tau pathology and neuroinflammation. While microgliosis is a well‐established feature of AD, the role of astrocytes is more nuanced. Consistent with emerging evidence of astrocytic dystrophy in the dentate gyrus of AD models, we observed a loss of GFAP+ astrocytes in P301L mice. TP2 treatment exerted a dual restorative effect: it dampened the neurotoxic activation of microglia while rescuing the number and the morphological integrity of astrocytes. This suggests that clearing intracellular Tau pathology can secondarily resolve the hostile neuroimmune microenvironment, creating a permissive state for neuronal recovery.

The robustness of TP2 is underscored by its efficacy across two mechanistically distinct models: the P301L transgenic model, which mimics familial tauopathy with mature tangles, and the AAV‐hTau‐N368 model, which recapitulates the toxicity of truncated Tau fragments often found in sporadic AD. In both models, systemic TP2 treatment translated molecular and cellular repairs into significant cognitive improvements. Although these findings establish therapeutic efficacy of TP2 across two distinct tauopathy models, the present study was not designed to determine whether TP2 provides superior in vivo safety or overall translational performance relative to peptide‐based DEPTACs. Direct head‐to‐head comparisons of pharmacokinetics, brain exposure, biodistribution, tolerability, and toxicology will be required to define the potential translational advantages of the small‐molecule modality. Additionally, the present LC–MS/MS analysis establishes measurable brain exposure of TP2 but does not resolve the mechanism by which it crosses the BBB. Although the structural origins of its two binding moieties provide a plausible context for the observed brain exposure, these structural precedents do not establish the actual transport route of the intact bifunctional molecule [40, 41, 42]. Dedicated studies will therefore be required to distinguish intrinsic passive permeability from transporter‐mediated uptake and efflux.

In conclusion, TP2 represents a paradigm shift in Tau‐targeting therapeutics, moving from “inhibition” or “degradation” to “correction” of the pathological state. Future studies will focus on optimizing the pharmacokinetic properties of TP2 and evaluating its efficacy in higher‐order animal models, paving the way for potential clinical translation.

## Author Contributions

**Fei Sun**, **Qiuzhi Zhou**, **Weixia Wang**, **Yue Xiao**, **Jingfen Su**, **Guanting Liu** and **Miaomiao Sun** performed the experiment and analyzed the data. **Yang Liu**, **Qingyuan Hu** designed, synthesized, and characterized the TP2. **Jian‐Zhi Wang**, **Ying Yang**, and **Hua Li** designed the study. Fei Sun, Yang Liu, and Qiuzhi Zhou wrote the manuscript. Jian‐Zhi Wang, and **Lixia Chen** reviewed the manuscript. All authors read and approved the final manuscript.

## Funding

This investigation was funded in parts by National Natural Science Foundation of China (82571635, 82230041, 82371436), the Fundamental Research Funds for the Central Universities (YCJJ20252412), Key Research and Development Program of Henan Province (241111310400).

## Ethics Approval and Consent to Participate

All animal‐related experiments were approved by the Animal Care and Use Committee of Huazhong University of Science and Technology. All procedures were conducted in accordance with the relevant guidelines and regulations. Consent to participate is not applicable.

## Conflicts of Interest

The authors declare no conflicts of interest.

## Supporting information




**Supporting File 1**: advs77088‐sup‐0001‐SuppMat.docx.

**Supporting File 2**: advs77088‐sup‐0002‐SuppMat.docx.

**Supporting File 3**: advs77088‐sup‐0003‐TableS1.docx.

## Data Availability

The data that support the findings of this study are available from the corresponding author upon reasonable request.
