# Supplementary material for: A Small‐Molecule DEPTAC Rescues Cognitive Deficits by Targeted Dephosphorylation of Pathological Tau
Source: Adv Sci (Weinh). 2026 Aug 11;13(61):e77088. doi: 10.1002/advs.77088 (PMC13460270; doi:10.1002/advs.77088)
Supplement: Supplementary file 2 [file ADVS-13-e77088-s002.docx]

**Supplementary information**

**Chemistry Section**

**Synthesis of compounds and their characterization**

**General information**

All reagents were procured from commercial sources and used without further purification. Silica gel GF254 and silica gel (200-300 mesh) were respectively used for thin-layer chromatography and column chromatography. NMR spectra were recorded on a Bruker AM-400 spectrometer (CDCl_3_ = 7.26 ppm for ^1^H NMR and 77.16 ppm for ^13^C NMR, DMSO-*d*_6_ = 2.50 ppm for ^1^H NMR and 39.52 ppm for ^13^C NMR). HRESIMS data were acquired using a Thermo Fisher LC-LTQ-Orbitrap XL spectrometer and an electrospray ionization and a hybrid quadrupole time-of-flight (q-TOF) mass spectrometer (model 6540, Agilent). Representative synthetic procedures for **TP2** were illustrated in **Figure 1**. Detailed reaction condition information and characterization data were described as following:

**Preparation of intermediate 2.**

2-Chlorophenothiazine (233.7 mg, 1 mmol) was added to a pear-shaped flask and dissolved in 5 mL of THF. The solution was cooled in an ice bath, followed by the addition of NaH (200 mg, 5 mmol). After stirring for 30 minutes, 1-bromo-3-chloropropane (787.2 mg, 5 mmol) was added dropwise to the mixture. The reaction was then heated to 65℃ and maintained for 5 hours. Upon completion, the solvent was removed under reduced pressure. The residue was dissolved in 20 mL of ethyl acetate, and the organic layer was washed three times with saturated brine. The organic phase was collected, dried over anhydrous Na_2_SO_4_, and purified via silica gel column chromatography. The target compound was obtained as a colorless oil with a yield of 80%. ^1^H NMR (400 MHz, CDCl_3_) *δ* 7.15 (ddd, *J* = 14.6, 7.5, 1.5 Hz, 2H), 7.02 (d, *J* = 8.1 Hz, 1H), 6.94 (td, *J* = 7.5, 1.1 Hz, 1H), 6.91 – 6.83 (m, 3H), 4.02 (t, *J* = 6.5 Hz, 2H), 3.64 (t, *J* = 6.1 Hz, 2H), 2.20 (d, *J* = 6.3 Hz, 2H).

**Preparation of intermediate 3.**

Intermediate 2 (70 mg, 0.226 mmol) was dissolved in 2 mL of acetonitrile, followed by the sequential addition of *N*-Boc-piperazine (63.2 mg, 0.339 mmol) and triethylamine (100 μL, 0.678 mmol). The reaction mixture was heated at 80°C for 4 hours. Upon completion, the solvent was removed under reduced pressure, and the resulting residue was dissolved in 10 mL of ethyl acetate. The organic layer was washed three times with saturated brine, collected, and dried over anhydrous Na_2_SO_4_. The crude product was purified by silica gel column chromatography to afford the target compound as a colorless oil (yield: 86%). ^1^H NMR (600 MHz, CDCl_3_) *δ* 7.08 – 7.00 (m, 2H), 6.90 (d, *J* = 8.0 Hz, 1H), 6.83 (td, *J* = 7.5, 1.1 Hz, 1H), 6.78 (td, *J* = 8.2, 1.5 Hz, 2H), 6.75 (d, *J* = 2.1 Hz, 1H), 3.81 (t, *J* = 6.7 Hz, 2H), 3.29 (t, *J* = 5.0 Hz, 4H), 2.36 (t, *J* = 6.9 Hz, 2H), 2.25 (t, *J* = 5.1 Hz, 4H), 1.82 (d, *J* = 6.9 Hz, 2H), 1.37 (s, 9H).

**Preparation of intermediate 4.**

Intermediate 3 was treated with an excess of HCl ethyl acetate solution under ice-cooling. The reaction mixture was stirred for 30 minutes. Upon completion, the excess reagent and solvent were removed under reduced pressure to afford intermediate 4 as a purple solid.

**Preparation of intermediate 6.**

3-Bromoisoquinoline (100 mg, 0.48 mmol) was dissolved in 2 mL of concentrated H_2_SO_4_. The solution was cooled in an ice bath, and KNO_3_ (58 mg, 0.58 mmol) was added slowly. After stirring for 30 minutes, the mixture was allowed to warm to room temperature and reacted for an additional 3 hours. The reaction mixture was then slowly poured into ice water, resulting in the precipitation of a pale yellow solid. The precipitate was collected by suction filtration, washed three times with water, and dried in an oven to afford the target product (yield: 90%). ^1^H NMR (400 MHz, DMSO-*d_6_*) *δ* 9.47 (s, 1H), 8.75 (dd, *J* = 7.7, 1.2 Hz, 1H), 8.66 (dd, *J* = 8.2, 1.3 Hz, 1H), 8.46 (s, 1H), 7.93 (t, *J* = 8.0 Hz, 1H).

**Preparation of intermediate 7.**

Intermediate 6 (100 mg, 0.4 mmol) was dissolved in 10 mL of glacial acetic acid. Under vigorous stirring, reduced iron powder (119 mg, 2 mmol) was added to the solution. Upon completion of the reaction, the mixture was filtered by suction filtration to remove the unreacted iron powder. The filtrate was diluted with water and extracted three times with ethyl acetate. The combined organic phases were washed three times with saturated brine, dried, and purified via silica gel column chromatography to afford the target compound as a buff-colored solid (yield: 76%). ^1^H NMR (400 MHz, DMSO-*d_6_*) *δ* 9.00 (s, 1H), 8.15 (s, 1H), 7.39 (t, *J* = 7.8 Hz, 1H), 7.28 (d, *J* = 8.1 Hz, 1H), 6.90 (dd, *J* = 7.6, 1.1 Hz, 1H), 6.09 (s, 2H).

**Preparation of intermediate 9.**

Intermediate 7 (80.5 mg, 0.37 mmol) was dissolved in 3 mL of *N*, *N*-dimethylformamide. Under ice-cooling, HATU (176.7 mg, 0.47 mmol), DIPEA (81 μL, 0.47 mmol), and intermediate 8 (86.3 mg, 0.31 mmol) were added sequentially. The reaction mixture was then allowed to warm to room temperature and stirred for an additional 2 hours. The mixture was diluted with 10 mL of water and extracted three times with ethyl acetate. The combined organic layers were washed three times with saturated brine, collected, and dried over anhydrous sodium sulfate. After purification by silica gel column chromatography, the target compound was obtained as a pale yellow solid (yield: 90%). ^1^H NMR (600 MHz, DMSO-*d_6_*) *δ* 9.87 (s, 1H), 9.13 (d, *J* = 0.8 Hz, 1H), 8.07 (d, *J* = 0.9 Hz, 1H), 7.95 (ddd, *J* = 7.7, 3.7, 1.1 Hz, 2H), 7.66 (t, *J* = 7.9 Hz, 1H), 4.20 (s, 2H), 3.83 (s, 2H), 3.71 – 3.67 (m, 2H), 3.62 – 3.58 (m, 2H), 3.56 – 3.48 (m, 4H), 1.32 (s, 9H).

**Preparation of intermediate 10.**

Intermediate 9 (41.6 mg, 0.086 mmol), 6-azaindole (12.0 mg, 0.103 mmol), Cu_2_O (0.24 mg, 0.002 mmol), BFMO (0.42 mg, 0.002 mmol), and K_3_PO_4_ (36 mg, 0.172 mmol) were added to a pear-shaped flask. After the system was purged with N_2_, 2 mL of anhydrous DMSO was added. The reaction mixture was then heated at 120°C overnight. After cooling to room temperature, the mixture was diluted with ethyl acetate and washed three times with saturated brine. The organic phase was collected, dried over anhydrous sodium sulfate, and purified by silica gel column chromatography to afford the target compound as a buff-colored solid (yield: 20%). ^1^H NMR (600 MHz, DMSO-*d_6_*) *δ* 10.03 (s, 1H), 9.44 (s, 1H), 8.36 (d, *J* = 3.3 Hz, 1H), 8.22 (s, 1H), 8.09 (d, *J* = 7.5 Hz, 1H), 8.03 (d, *J* = 8.2 Hz, 1H), 7.80 (s, 1H), 7.67 (t, *J* = 7.8 Hz, 1H), 6.91 (d, *J* = 3.3 Hz, 1H), 3.56 (s, 3H), 2.54 (t, *J* = 7.4 Hz, 2H), 2.25 (t, *J* = 7.4 Hz, 2H), 1.69 (d, *J* = 7.4 Hz, 2H), 1.47 (d, *J* = 6.8, 6.4 Hz, 2H), 1.37 (d, *J* = 7.2 Hz, 2H), 1.33 – 1.27 (m, 2H), 1.25 – 1.20 (m, 4H).

**Preparation of intermediate 11.**

Intermediate 10 was dissolved in dichloromethane. The solution was cooled in an ice bath, and TFA (10 equivalents) was added. The reaction mixture was then allowed to warm to room temperature and stirred for 3 hours. Upon completion, the solvent and excess TFA were removed by distillation under reduced pressure to afford the product (yield: 96%).

**Synthesis of target compound TP2.**

Intermediate 11 (5.2 mg, 0.0112 mmol) was dissolved in 2 mL of dichloromethane. Under ice-cooling, EDCI (4.3 mg, 0.022 mmol), HOBt (3.0 mg, 0.022 mmol), DIPEA (78 μL, 0.045 mmol), and intermediate 4 (5.3 mg, 0.013 mmol) were added sequentially. The mixture was stirred for 30 minutes, then allowed to warm to room temperature and reacted for 2 hours. The reaction mixture was diluted with 10 mL of dichloromethane and washed three times with water. The organic phase was collected, dried over anhydrous Na_2_SO_4_, and purified by silica gel column chromatography to afford the target compound as a pale yellow solid with a yield of 67%. ^1^H NMR (600 MHz, DMSO-*d*_6_) *δ* 9.99 (s, 1H), 9.67 (s, 1H), 9.43 (s, 1H), 8.31 (d, *J* = 3.4 Hz, 1H), 8.11 (s, 1H), 8.07 (d, *J* = 8.2 Hz, 1H), 8.02 (d, *J* = 7.4 Hz, 1H), 7.95 (d, *J* = 7.7 Hz, 2H), 7.51 (t, *J* = 7.0 Hz, 2H), 7.38 (dd, *J* = 15.2, 8.0 Hz, 2H), 7.19 (t, *J* = 7.9 Hz, 1H), 7.15 – 7.10 (m, 2H), 7.03 (d, *J* = 8.0 Hz, 2H), 6.99 – 6.94 (m, 2H), 6.88 (t, *J* = 2.5 Hz, 1H), 4.31 – 4.28 (m, 2H), 3.95 – 3.92 (m, 2H), 3.88 (d, *J* = 6.8 Hz, 2H), 3.78 (d, *J* = 4.8 Hz, 2H), 3.66 (s, 2H), 3.52 (d, *J* = 4.4 Hz, 2H), 3.28 (s, 4H), 3.19 (s, 2H), 2.33 (d, *J* = 6.7 Hz, 2H), 2.22 (d, *J* = 10.4 Hz, 4H), 1.76 – 1.70 (m, 2H). ^13^C NMR (150 MHz, DMSO-*d_6_*) *δ* 169.88, 169.43, 150.14, 149.16, 146.22, 143.71, 140.75, 136.17, 135.55, 134.05, 132.62, 132.22, 129.35, 128.27, 127.78, 127.70, 127.62, 127.47, 126.15, 123.96, 123.32, 123.30, 123.13, 123.11, 119.37, 117.00, 116.51, 116.42, 108.36, 108.26, 70.35, 70.30, 70.06, 69.59, 69.58, 69.38, 53.66, 52.86, 49.51, 45.69, 24.70. HRMS (ESI^+^): *m/z* calculated for C_43_H_44_ClN_7_O_5_S [M + H]^+^, 806.2886; found, 806.2888.

**Characterization data** **of compounds**

^1^H NMR spectra of **intermediate 2**

^1^H NMR spectra of **intermediate 3**

^1^H NMR spectra of **intermediate 6**

^1^H NMR spectra of **intermediate 7**

^1^H NMR spectra of **intermediate 9**

^1^H NMR spectra of **intermediate 10**

^1^H NMR spectra of **TP2**

^13^C NMR spectra of **TP2**

HRMS spectrum of **TP2**

HPLC analysis of **TP2**

by YMC-Triart C18 column (250 mm × 4.6 mm, 5 μm; MeOH:H_2_O = 70:30, 0.8 mL/min, 232 nm.)
