# Supplementary material for: A Small‐Molecule DEPTAC Rescues Cognitive Deficits by Targeted Dephosphorylation of Pathological Tau
Source: Adv Sci (Weinh). 2026 Aug 11:e77088. Online ahead of print. doi: 10.1002/advs.77088 (PMC13460270; doi:10.1002/advs.77088)
Supplement: Supplementary file 2 [file ADVS-9999-e77088-s002.docx]

**Supplemental Figures**

**Supplementary Figure 1. TP2 does not interfere with Tau-induced microtubule assembly in vitro.**

(A) Microtubule assembly was monitored by turbidity (A340) at 37 °C every 3 min over 45 min. Tubulin (10 μM) was incubated alone or with GST-Tau (5 μM), TP2 (20 μM), or both. TP2 alone did not induce assembly, and co-incubation of TP2 with GST-Tau did not alter the GST-Tau–induced assembly curve.

(B–E) Kinetic parameters quantified from (A): lag time (B), maximum assembly rate (C), extent of assembly (D), and area under the curve (E). TP2 had no significant effect on any GST-Tau–induced parameter (unpaired Student's t-test, ns). n = 3 independent experiments. Data are presented as mean ± SEM.

**Supplementary Figure 2. Comparison of TP2 and the peptide-based DEPTAC D16 in reducing Tau phosphorylation in primary neurons.**

(A) Representative Western blots of primary rat hippocampal neurons treated with TP2 or D16 at the indicated concentrations (0, 5, 10, 20 μM) for 24 h, showing p-Tau (AT8), total Tau (Tau5), and β-actin.

(B) Representative Western blots from the washout experiment. Neurons were treated with 5 μM TP2 or 20 μM D16 for 24 h, after which the medium was replaced with drug-free medium. Lysates were then collected at 1, 3, 5, and 7 days after washout, with vehicle-treated neurons (Veh) included as a control. Blots show AT8, Tau5, and β-actin. (C-E) Quantification of the relative band intensity normalized to β-actin for AT8 (C) and Tau5 (D), and the corresponding AT8/Tau5 ratio (E) in the concentration-gradient experiment. TP2 produced a marked reduction in AT8 and a lower AT8/Tau5 ratio at 5 μM, whereas D16 required 20 μM to achieve a comparable effect. At 5 and 10 μM, TP2 induced greater reductions in AT8 and lower AT8/Tau5 ratios than D16. n = 3, two-way ANOVA with Šídák's multiple comparisons test, *p < 0.05, **p < 0.01, ***p < 0.001, ****p < 0.0001 TP2 vs D16.

(F-H) Quantification of the relative band intensity normalized to β-actin for AT8 (F) and Tau5 (G), and the corresponding AT8/Tau5 ratio (H) in the washout experiment. After drug removal, the reduction in AT8 and in the AT8/Tau5 ratio persisted longer in the TP2 group than in the D16 group, whereas Tau5 levels did not differ significantly between the two treatment groups during the washout period. n = 3, two-way ANOVA with Šídák's multiple comparisons test, **p < 0.01, ****p < 0.0001 TP2 vs D16.

**Supplementary Figure 3. TP2-induced dephosphorylation renders Tau susceptible to ubiquitin–proteasome-dependent clearance.**

(A) Representative Western blots from a cycloheximide (CHX) chase assay. HEK293-Tau cells were treated with 100 μg/mL CHX in the presence of vehicle or 10 μM TP2. Cells were collected at 0, 6, 12, and 24 h, and AT8, Tau5, and β-actin were detected by Western blotting.

(B) Representative Western blots showing AT8, Tau5, and β-actin in HEK293-Tau co-treated with 10 μM TP2 and 10 μM proteasome inhibitor MG132 or 100 nM autophagy–lysosome inhibitor bafilomycin A1 (BafA1) for 8 h.

(C–E) Quantification of the relative intensity of AT8 (C), Tau5 (D), and the AT8/Tau5 ratio (E) from (A), normalized to β-actin and expressed relative to the 0 h time point. TP2 accelerated the decline of Tau5 during the CHX chase, suggesting enhanced Tau degradation following TP2 treatment. n = 3, two-way ANOVA with Šídák's post-hoc test, *p < 0.05, **p < 0.01, ***p < 0.001 CHX vs CHX+TP2.

(F–H) Quantification of the relative intensity of AT8 (F), Tau5 (G), and the AT8/Tau5 ratio (H) from (B), normalized to β-actin. The TP2-induced reduction of Tau5 was abolished by MG132 but not by BafA1, whereas the reduction in AT8 persisted in the presence of MG132. These results indicate that TP2-induced dephosphorylation precedes the subsequent proteasome-dependent reduction in total Tau. n = 3, one-way ANOVA with Tukey's post-hoc test, *p < 0.05, ***p < 0.001, ****p < 0.0001.

(I, J) Tau ubiquitination assay. HEK293-Tau were treated with vehicle or 10 μM TP2 for 4 h, followed by the addition of 10 μM MG132 for an additional 4 h to stabilize ubiquitinated species. Tau was then immunoprecipitated under denaturing conditions with Tau5 antibody and immunoblotted for ubiquitin (IB: Ub); immunoprecipitated Tau (IP: Tau5) and input Tau (Input: Tau5). Representative blots (I) and quantification of the normalized relative ubiquitination intensity (J) show that TP2 increased the ubiquitination of Tau compared with the vehicle group. n = 3, unpaired Student's t-test, **p < 0.01. Data are presented as mean ± SEM.

**Supplementary Figure 4. LC–MS/MS detection of TP2 in brain and plasma following intravenous administration.**

Eight-week-old male C57BL/6J mice received a single intravenous tail-vein injection of vehicle or TP2 (2.5 mg/kg). Thirty minutes after administration, blood was collected for plasma preparation, followed by thorough saline perfusion before brain collection. Representative multiple-reaction-monitoring chromatograms are shown for TP2 (m/z 806.2 → 419.0) and the internal standard glibenclamide (IS; m/z 494.2 → 369.2). (A, B) Brain from a vehicle-treated mouse, analyzed in the TP2 and IS channels, respectively; (C, D) brain from a TP2-treated mouse; (E, F) plasma from a vehicle-treated mouse; and (G, H) plasma from a TP2-treated mouse. TP2 was detected in the brain and plasma of TP2-treated mice, with retention times of approximately 1.26 min, whereas the IS eluted at approximately 1.90 min.

**Supplementary Figure 5. TP2 reduces Tau phosphorylation in the cortical lysates of P301L mice.**

(A) Representative Western blots of cortical lysates from WT, vehicle-treated P301L, and TP2-treated P301L mice showing p-Tau (pT181, pS199, pS396, pS404, AT8) and β-actin. The blots clearly resolve two distinct species: the upper band corresponds to exogenous human P301L Tau (~70 kDa) and the lower band corresponds to endogenous mouse Tau (~55 kDa).

(B) Quantification of the endogenous mouse Tau normalized to β-actin (~55 kDa). TP2 treatment reduced the phosphorylation levels of endogenous Tau compared to the vehicle-treated P301L group. n = 3, one-way ANOVA with Tukey's post-hoc test, **p* < 0.05, ***p* < 0.01.

(C) Quantification of the exogenous human P301L Tau normalized to β-actin (~70 kDa). TP2 treatment indicated a decreasing trend in the phosphorylation of exogenous human Tau, although these differences did not reach statistical significance. n = 3, unpaired Student's t-test. Data are presented as mean ± SEM.

**Supplementary Figure 6. TP2 decreases insoluble Tau aggregates in the hippocampus of P301L mice.**

(A) Representative Western blots of the RIPA-insoluble fraction extracted from WT, vehicle-treated P301L, and TP2-treated P301L mice showing p-Tau (pT181, pS199, pS396, pS404, AT8) and total Tau (Tau5).

(B) Quantification of the insoluble endogenous mouse Tau (~55 kDa) normalized to β-actin revealed that TP2 treatment effectively reduced the accumulation of aggregated endogenous Tau species compared to the vehicle-treated P301L group. n = 3, one-way ANOVA with Tukey's post-hoc test, **p* < 0.05.

(C) Quantification of the insoluble exogenous human P301L Tau (~70 kDa) normalized to β-actin showing a significant decrease in insoluble human Tau aggregates following TP2 administration. n = 3, unpaired Student's t-test, **p* < 0.05 vs Vehicle. Data are presented as mean ± SEM.
