# Supplementary material for: A Small‐Molecule DEPTAC Rescues Cognitive Deficits by Targeted Dephosphorylation of Pathological Tau
Source: Adv Sci (Weinh). 2026 Aug 11:e77088. Online ahead of print. doi: 10.1002/advs.77088 (PMC13460270; doi:10.1002/advs.77088)
Supplement: Supplementary file 3 [file ADVS-9999-e77088-s003.docx]

**Supplementary Table 1. TP2 concentrations in plasma and brain after vehicle or TP2 administration.**

| **Matrix** | **Vehicle control** | **TP2-treated Mouse 1** | **TP2-treated Mouse 2** | **TP2-treated Mouse 3** | **TP2-treated Mean ± SD** | **Brain-to-plasma ratio** |
| --- | --- | --- | --- | --- | --- | --- |
| Plasma (ng/mL) | ND | 125 | 134 | 120 | 126 ± 7 | — |
| Brain (ng/g) | ND | 4.52 | 3.93 | 4.53 | 4.33 ± 0.34 | 0.034 |

ND, no TP2 peak detected in the vehicle-control sample. Concentrations are expressed as ng/mL for plasma and ng/g for brain. Mean ± SD and the brain-to-plasma ratio were calculated from the three TP2-treated mice.
